# Rapidly Growing *Mycobacterium* Species: The Long and Winding Road from Tuberculosis Vaccines to Potent Stress-Resilience Agents

**DOI:** 10.3390/ijms222312938

**Published:** 2021-11-29

**Authors:** Mattia Amoroso, Dominik Langgartner, Christopher A. Lowry, Stefan O. Reber

**Affiliations:** 1 Laboratory for Molecular Psychosomatics, Department of Psychosomatic Medicine and Psychotherapy, University of Ulm, 89081 Ulm, Germany; mattia.amoroso@uni-ulm.de (M.A.); dominik.langgartner@uni-ulm.de (D.L.); 2 Department of Integrative Physiology, Center for Neuroscience and Center for Microbial Exploration, University of Colorado Boulder, Boulder, CO 80309, USA; christopher.lowry@colorado.edu; 3 Department of Physical Medicine and Rehabilitation and Center for Neuroscience, University of Colorado Anschutz Medical Campus, Aurora, CO 80045, USA; 4 Veterans Health Administration, Rocky Mountain Mental Illness Research Education and Clinical Center (MIRECC), The Rocky Mountain Regional Veterans Affairs Medical Center (RMRVAMC), Aurora, CO 80045, USA; 5 Military and Veteran Microbiome: Consortium for Research and Education (MVM-CoRE), Aurora, CO 80045, USA; 6 Senior Fellow, inVIVO Planetary Health, of the Worldwide Universities Network (WUN), West New York, NJ 07093, USA

**Keywords:** immunoregulation, inflammation, *Mycobacterium kyogaense*, *Mycobacterium vaccae*, old friends, stress-associated disorders, stress resilience

## Abstract

Inflammatory diseases and stressor-related psychiatric disorders, for which inflammation is a risk factor, are increasing in modern Western societies. Recent studies suggest that immunoregulatory approaches are a promising tool in reducing the risk of suffering from such disorders. Specifically, the environmental saprophyte *Mycobacterium vaccae* National Collection of Type Cultures (NCTC) 11659 has recently gained attention for the prevention and treatment of stress-related psychiatric disorders. However, effective use requires a sophisticated understanding of the effects of *M. vaccae* NCTC 11659 and related rapidly growing mycobacteria (RGMs) on microbiome–gut–immune–brain interactions. This historical narrative review is intended as a first step in exploring these mechanisms and provides an overview of preclinical and clinical studies on *M. vaccae* NCTC 11659 and related RGMs. The overall objective of this review article is to increase the comprehension of, and interest in, the mechanisms through which *M. vaccae* NCTC 11659 and related RGMs promote stress resilience, with the intention of fostering novel clinical strategies for the prevention and treatment of stressor-related disorders.

## 1. Introduction

### 1.1. The “Old Friends” Hypothesis: A Biological Concept to Explain the Increasing Prevalence Rates of Stress-Associated Inflammatory Disorders in Modern Urban Societies

The prevalence of many stress-associated somatic disorders including allergies [1] and autoimmune diseases [2,3,4,5] as well as mental pathologies such as depression and posttraumatic stress disorder (PTSD) [6] has increased over the past decades in Westernized countries, overall representing a serious health and economic burden for our modern society. Although the mechanisms underlying both development and progression of these stress-associated disorders are not fully understood, and, consequently, prevention and treatment options for many of these disorders are still limited, a common feature of these disorders is a dysregulated immune system and increased inflammation [7]. As many of these stress-associated disorders are further characterized by a compromised regulatory T cell (Treg) compartment [8,9,10], a failure of immunoregulation has been hypothesized to be involved in promoting an over-reacting immune system, thus, predisposing an individual to disease development. Thus, anti-inflammatory and immunoregulatory approaches might be a useful tool in prevention and treatment of stress-related disorders. According to the “old friends” hypothesis, deficits in immunoregulation are due to reduced contact with harmless microorganisms that accompanied mammalian evolution in high abundance and had to be tolerated by an individual’s immune system to avoid damage caused by chronic inflammatory processes [11]. Interestingly, these “old friend” organisms promote their own survival and, as a beneficial side effect, the health of their host by facilitating immunoregulation. “Old friends” fall into three main categories: (1) microorganisms associated with “old infections” that were common in human evolutionary past (helminths, *Salmonella*, *Helicobacter pylori* [12,13]); (2) microorganisms that were part of the human microbiota (gut, airway, skin, genitourinary, oropharyngeal; [14,15,16,17]); and (3) harmless microorganisms from the natural environment in water, air, and soil with which humans inevitably had regular contact (reviewed in [18,19]). Two such microorganisms attracting attention for their immunoregulatory effects are *Mycobacterium vaccae* National Collection of Type Cultures 11659 (*M. vaccae* NCTC 11659) and *Mycobacterium vaccae* American Type Culture Collection 15483 ^Typestrain^ (*M. vaccae* ATCC 15483^T^) [20,21,22,23,24,25,26,27,28,29,30,31,32,33]. In the current article, we aim to provide a narrative review of the research history of these two immunoregulatory mycobacteria in a chronological way, starting with the first observational studies on their promising effects as tuberculosis (TB) vaccines up to the most recent studies indicating that these “old friends” have stress-protective effects and promote stress resilience. Special emphasis is given to the cellular and molecular mechanisms known so far to mediate the effects of these rapidly growing mycobacteria (RGMs) including the recently discovered 10(*Z*)-hexadecenoic acid (10(*Z*)-HDA), a free fatty acid synthetized by *M. vaccae* NCTC 11659 that mediates its anti-inflammatory effects via enhancement of peroxisome proliferator-activated receptor alpha (PPARα) signaling. Finally, the effectiveness of different routes of *M. vaccae* NCTC 11659 and *M. vaccae* ATCC 15483^T^ administration and possible strategies to increase it are discussed. Important to note is that *M. vaccae* NCTC 11659 (Colección Española de Cultivos Tipo (CECT) 9646^T^; DSMZ-Deutsche Sammlung von Mikroorganismen und Zellkulturen GmbH (DSM) 107316^T^) was reclassified in 2018 as *Mycobacterium kyogaense* sp. nov. NCTC 11659^T^ [34,35]. However, to avoid any confusion as this *Mycobacterium* strain has been referenced in many previously published articles including our own [6,20,21,32,33] as *M. vaccae* NCTC 11659, we keep this nomenclature consistent and refer to this *Mycobacterium* strain in the current review article as *M. vaccae* NCTC 11659 (please see Table 1 for alternative designations and different preparations and production processes of *M. vaccae* NCTC 11659). When discussing studies investigating the effects of the *M. vaccae* type strain, we refer to it as *M. vaccae* (ATCC 15483^T^; DSM 43292^T^; NCTC 10916^T^), and to *M. vaccae* when the exact *M. vaccae* strain was not further specified in the original articles. An overview of the exact nomenclature, preparation, and production details and dose of the *Mycobacterium* species/strain used in each study discussed in the current review article is provided in Table 1, Table 2, Table 3 and Table 4.

### 1.2. Mycobacterium vaccae NCTC 11659: General Information

*M. vaccae* NCTC 11659 is a rapidly growing, aerobic, Gram-positive, acid-alcohol-fast, rod-shaped soil saprophyte, which forms rough yellow pigmented colonies in culture [34]. *Mycobacterium* strains can be ubiquitously found in water and soil as well as in manufactured water distribution systems [77,78] and are the dominant taxa in municipal showerheads [79]. Although mycobacteria are not normally found in the human gut microbiome, they are abundant in the human oral cavity (buccal mucosa and dental plaque) and upper respiratory tract (nostrils and oropharynx) [80]. Studies comparing the airway microbiome in urban versus rural children in Denmark have found greater abundance of mycobacteria in rural children versus urban children at three months of age [81]. Although no evidence of pathogenicity of *M. vaccae* NCTC 11659 has ever been shown, in 1996, a non-identified strain of *M. vaccae* was for the first time reported to cause non-severe infections in immunocompromised individuals [82]. More recently, another non-identified strain of *M. vaccae* was reported to cause catheter-related sepsis in a patient with follicular non-Hodgkin lymphoma [83]. Its name is derived from the Latin word for cow, “*vacca*”, as the first discovered strain was isolated from cow dung in Austria [50] and known in the literature under the designations ATCC 15483^T^, DSM 43292^T^, and NCTC 10916^T^ [34,84]. On the other hand, *M. vaccae* NCTC 11659 was first isolated from the mud of Lake Kyoga in Uganda by Stanford and Paul [85]. Although incorrectly classified as *M. vaccae* NCTC 11659, data based on 16S rRNA gene and genome sequencing provided a rationale for its reclassification as *M. kyogaense* sp. nov [34]. This strain was originally grown on Sauton’s medium solidified with 1.5% agar at 32 °C, but showed optimal growth at 37 °C on glucose, yeast, and malt (GYM) agar, Middlebrook (MB) 7H10 agar, and proteose peptone-meat extract-glycerol (PMG) agar [34,86]. At the end of the period of logarithmic growth (about seven days), the bacterial growth is usually scraped from the surface of the medium, weighed, and suspended in borate-buffered saline (BBS) at a concentration of 10 mg of wet weight/mL [34]. Heat-killed preparations of *M. vaccae* NCTC 11659 can be prepared by autoclaving in BBS at 121 °C for 15 min [87].

Several lines of evidence have shown that a heat-killed preparation of *M. vaccae* NCTC 11659 had remarkable immunomodulatory and, thus, health promoting properties in both preclinical and clinical studies [88]. This is indicated by its beneficial effects against infectious diseases such as leprosy and TB [85,87,89], chronic inflammatory disorders such as asthma [40,69,90], colitis [32,33] as well as various forms of cancer in humans [66,68,71,91,92,93,94,95]. Strikingly, *M. vaccae* NCTC 11659 was also protective in animal models of inescapable stress, fear conditioning, post-operative cognitive impairment in aged rats, models of “two hit” stressors involving sleep deprivation or chronic disruption of rhythms and social defeat, and chronic psychosocial stress [20,21,22,23,24,25,26,27,28,29,30,31,32,33]. The effects of *M. vaccae* NCTC 11659 in all of the above-referenced studies are discussed in a more detailed manner in the following sections of this review article. Both *M. vaccae* NCTC 11659 [88] and *M. vaccae* ATCC 15483^T^ [77] have peculiar immune modulating properties that are hypothesized to depend on the extraordinary complexity of the cell envelope, a feature characteristic for the whole *Mycobacterium* genus [96,97,98,99]. The envelope of mycobacteria adopts a unique dual membrane structure with a waxy outer membrane rich in mycolic acids and free lipids [100,101], a polysaccharide cell wall, and an inner cytoplasmic membrane (reviewed in [96,97,98]). Molecules in the outer membrane can be recognized by macrophages and dendritic cells (DCs) through pattern recognition receptors (PRRs) such as Toll-like receptors (TLRs), nucleotide-binding oligomerization domain (NOD)-like receptors (NLRs), and C-type lectin receptors (CLRs) (reviewed in [98]). These interactions influence DC maturation and, as a consequence, modulate subsequent immune responses, for example, by promoting naïve T cells to develop either into classic T helper (Th) 1 and Th2 cells and, in the case of *M. vaccae* NCTC 11659, into Th1 cells [71,102,103] and Tregs [33,40,88].

Considering *M. vaccae* ATCC 15483^T^, it can form both rough and smooth colonies in solid culture, and the shift from smooth to rough colony type occurs at temperatures above 30 °C [53]. Interestingly, while the rough colonies induced a Th1 response upon subcutaneous (s.c.) injection in mice, as shown by increased interferon (IFN)γ and interleukin (IL)-12 (p40) production, the smooth variant induced a significantly weaker production of the above-mentioned cytokines, but a higher IL-10 production instead [53]. Analyzing the lipid profiles of the two colony variants, the authors identified a long-chain saturated fatty acid polyester of estolide-like structure that is produced by the smooth, but not rough, *M. vaccae* ATCC 15483^T^ colony variant. This substance, named RC by the authors (i.e., “red color”, as it stained red with anthrone), seems to be the putative substance that explains the differential immune-polarizing properties of the two kinds of *M. vaccae* ATCC 15483^T^ colonies [53].

The immunomodulatory properties of *M. vaccae* NCTC 11659 seem to be best retained when the microorganism is heat-killed by autoclaving in BBS. In turn, *M. vaccae* NCTC 11659 autoclaved in phosphate-buffered saline (PBS) is much less effective, potentially because autoclaving in a borate solution breaks down proteins into short amino acid chains, which are stably preserved [88,104]. Autoclaving *M. vaccae* NCTC 11659 in PBS is also considered to reduce the amount of the so called group I antigens [88], which are common among the whole *Mycobacterium* genus and can suppress inflammation given their high homology with some heat shock proteins located in the mitochondria of eukaryotic cells, namely the heat-shock protein (hsp) 60 [105,106] and hsp70 [107,108]. Autoclaving *M. vaccae* NCTC 11659 in BBS ensures appropriate presentation of the amino acid chains of those antigens to naïve T cells, thereby resulting in a more stable product as opposed to autoclaving in PBS [88,103] or killing by exposing the bacterium to ^60^Co [109].

## 2. History of *Mycobacterium vaccae* NCTC 11659 Research

The history of *M. vaccae* NCTC 11659 research is strongly linked to the efforts to find an effective TB and leprosy vaccine. To date, Bacillus Calmette–Guèrin (BCG) remains the only effective vaccine against TB available for human use [110]. BCG was developed at the beginning of the 20th century as a suspension of live, attenuated *Mycobacterium bovis* bacilli isolated from a calf believed to be infected with the bovine form of TB, and was found to protect children from active TB [111]. With respect to the underlying mechanisms, it has been shown in BCG-vaccinated mice that the immune response against *Mycobacterium tuberculosis* is characterized by an increased accumulation of effector T cells at the site of active infection as well as increased production of Th1 cytokines, leading to restricted growth of the bacilli [112,113]. Of note, the immunotherapeutic efficacy of BCG seems to rely on both cluster of differentiation (CD) 4^+^ and CD8^+^ T cell subsets, as depletion of either cell type results in the failure of BCG therapy [114]. Both CD4^+^ and CD8^+^ T cell subsets in turn are dependent on the enhanced survival and prolonged lifespan of DCs following BCG injection, which are achieved through reduced rates of apoptosis [115]. However, BCG vaccination results in variable degrees of protection against TB and leprosy [116,117], being very effective in certain areas such as Uganda [85] and poorly effective in others such as India [118] and Myanmar [119]. The reason behind the geography-dependent effects of BCG vaccination was believed to be environmental in nature. Interestingly, in the search for an environmental factor that could explain the high success rate of BCG vaccination in Uganda, Dr. John Stanford noticed that the mud in and around Lake Kyoga in Uganda was particularly rich in *M. vaccae* NCTC 11659 [85,120,121], while *Mycobacterium scrofulaceum* was abundantly present in Myanmar [119]. Some years later, Stanford and colleagues could indeed show that *M. vaccae* NCTC 11659 enhances the protective post-BCG immune responses in Uganda, while *M. scrofulaceum* blocks them in Myanmar [119].

In more detail, the geography-dependent effectiveness of BCG vaccination against TB seems to be dependent, among other factors, on the environmental mycobacterial species present in the areas where studies on BCG were conducted. Rook and colleagues postulated that the latter was due to the two types of cell-mediated immune responses generally driven by *M. tuberculosis* infection [122]. The first, the “Koch-type”, initially described by Robert Koch in guinea pigs at the end of the 19th century [123,124] develops 4–6 weeks after *M. tuberculosis* infection, as indicated by a positive tuberculin skin test, and is characterized by a mixed Th1 and Th2 immune response, promoting the necrotizing effects of tumor necrosis factor (TNF) in the presence of the Th2 cytokines IL-4 and IL-5 and immunoglobulin E (IgE) [125,126]. The second cell-mediated immune response, the “Listeria-type” [127], occurs within days following *M. tuberculosis* infection and is characterized by the appearance of macrophage-activating Th1 lymphocytes [128]. The term “Listeria-type” immune response has been coined by George Mackaness [129] investigating the immune response against *Listeria monocytogenes* in mice. In contrast to the “Koch-type” response, this response was not accompanied by necrosis and strongly correlated with protection against *M. tuberculosis* and *Mycobacterium leprae* [130]. Interestingly, different species of mycobacteria have been demonstrated in animal models to induce these two types of immune responses, characterized by either an activation of Th1/Th2 (“Koch-type”) or solely Th1 (“Listeria-type”) immune response, to varying degrees [122,131]. For instance, while some mycobacterial strains induce only the “Listeria-type” of response, others promote only the “Koch-type”. Therefore, given the influence of different mycobacterial species on cellular-mediated immune responses, the predominant immune response to BCG vaccination found in a particular geographic region and, thus, the success rate of the BCG vaccine to protect individuals from *M. tuberculosis*-induced TB, was hypothesized to strongly depend on the environmental abundance of particular mycobacterial species as well as the relative abundances of different mycobacterial strains in the environment [119,122]. From this point of view, Uganda turned out to be an ideal place for testing this hypothesis, as environmental mycobacteria vary in their individual abundance and general composition from place to place, depending on the humidity and pH of the soil from where they are retrieved. In fact, Uganda is a country with a great variety of environmental conditions including forests, grasslands, and both acid and alkaline swamplands that guarantees the optimal habitat for a variety of mycobacteria species [132]. Among the many species isolated, *M. avium, M. nonchromogenicum, M. engbaekii, M. gordonae, M. fortuitum, M. vaccae, M. neoaurum,* and *M. kansasii* were the most abundant [132].

In these studies, it turned out that *M. vaccae* NCTC 11659 can only induce a “Listeria-type” response [133,134], which, if pre-existing, markedly boosts the immune response toward the BCG vaccine and thereby enhances the capacity of an organism to recognize and control further environmental mycobacterial species [119]. Thus, these data support the hypothesis that the high success rates of BCG vaccination against TB in particular areas of Uganda are due to the high environmental abundance of *M. vaccae* NCTC 11659 and related strains [85]. The “Listeria-type” response induced by *M. vaccae* NCTC 11659 thereby promotes the ability of the organism to induce a Th1 response; the Th1-polarizing effects of *M. vaccae* NCTC 11659 were then demonstrated in studies in mice [70,90,135], humans [67,136,137], and in in vitro studies employing human DCs [46]. This is thought to counteract the pathological shift toward the detrimental *M. tuberculosis*-induced “Koch-type” immune response, which prevents clearance of pathogen-infected cells [74]. Interestingly, the ability of *M. vaccae* NCTC 11659 to shift the immune response from a Th2 toward a Th1 response depends on the dose of *M. vaccae* NCTC 11659 administered. While a low-dose of *M. vaccae* NCTC 11659 (10^7^ bacterial cells given subcutaneously in mice) induces a protective Th1 response, a high-dose of *M. vaccae* NCTC 11659 (10^9^ bacterial cells) promotes a mixed Th1/Th2 response with detrimental effects for infection with *M. tuberculosis* [138]. These data are in agreement with previous findings of increased pathogenicity of TB when a mixed Th1/Th2, rather than a pure Th1 immune response, ensues after *M. tuberculosis* infection [139,140]. In contrast, *M. scrofulaceum* can induce responses of either the “Koch-“ or the “Listeria-type” depending on the frequency with which it and other environmental mycobacterial species are encountered [119]. Thus, the high amount of “Koch-type” reactions found in children in Myanmar, together with the high abundance of *M. scrofulaceum* present in the environment, may explain the low rate of success of BCG vaccination in Myanmar [141]. Following these early studies, many more observational, and later also mechanistic, studies were conducted to investigate the protective effects of *M. vaccae* NCTC 11659 in a variety of contexts. These studies are discussed in a chronological way in the following sections.

### 2.1. Observational Studies on the Protective Effects of M. vaccae NCTC 11659 and M. vaccae ATCC 15483^T^: Chronological Evidence

The following section summarizes the relevant literature on *M. vaccae* NCTC 11659 immunotherapy in the context of several conditions including TB, leprosy, psoriasis, dermatitis, asthma, and cancer. Specifically, the focus will be on observational studies (i.e., clinical trials in humans, and on the final outcomes of different formulations of *M. vaccae* NCTC 11659 in the progression of the above-mentioned pathologies. We subsequently focus on the cellular and molecular mechanisms of action of *M. vaccae* NCTC 11659. In a similar way, studies employing *M. vaccae* ATCC 15483^T^ will also be mentioned and discussed.

#### 2.1.1. *M. vaccae* NCTC 11659 and TB

##### Single Intradermal *M. vaccae* NCTC 11659 Administration as an Adjunct Therapy for First-Line Drug Therapy for Treatment of TB

TB represents a global health problem that is further aggravated by malnutrition and poor hygiene in developing countries, and is one of the most common co-infections and causes of death among human immunodeficiency virus (HIV)-infected individuals [142]. Moreover, certain *M. tuberculosis* strains are known to cause difficult-to-treat infections such as multi-drug-resistant [143], extremely drug resistant, and totally drug resistant TB [96,144], overall generating high socioeconomic burden [43]. Therefore, there is a clear unmet need for developing novel and effective drugs for the prevention and treatment of TB. Noteworthy, immune-based interventions employing *M. vaccae* NCTC 11659 as an adjunct therapy to standard anti-TB treatment have shown promising results in this context. *M. tuberculosis* is an intracellular pathogen, and it can express hsps that are highly cross-reactive with the hsps of the host [145]. The inflammatory response directed against the hsps of *M. tuberculosis* can result in the production of a spectrum of autoantibodies similar to what is seen in rheumatoid arthritis patients [146]. Although the main target of *M. tuberculosis* are phagocytic cells, in vitro studies have indicated that it can also infect other cell types [147,148]. Interestingly, infected endothelial cells and fibroblasts can only rarely be detected in vivo in histological sections of tissues. One possible explanation might be that in vivo these cells are killed very rapidly, which is supported by the observation that cells containing *M. tuberculosis* are exquisitely sensitive to killing by TNF [147,148]. Therefore, macrophages infected in vitro may be killed by their own production of TNF, while non-macrophage cells survive in vitro in the absence of TNF, but are rapidly killed in vivo since TNF is abundant in TB lesions [149]. As above-mentioned, the immune response against *M. tuberculosis* can further promote necrosis of the infected tissue through the combined action of TNF [150], type 2 cytokines IL-4 and IL-5, and IgE [125,126,151]. Studies in mice [138] revealed that *M. vaccae* NCTC 11659 (10^7^ bacilli) induces a strong Th1 immune response, activating infected macrophages to kill bacteria surviving in their phagosomes as well as promoting clearance of these infected macrophages by CD8^+^ cytotoxic T lymphocyte (CTL), together ameliorating TB pathogenesis [152,153]. On the other hand, a higher dose (10^9^ bacilli) induces a mixed Th1/Th2 response with detrimental effects against tuberculosis [138]. Based on these animal studies, several clinical trials were initiated in areas where TB is still endemic.

In a clinical study conducted in 1999 in Argentina by Dlugovitzky and colleagues [55], individuals with TB received a single intradermal injection of heat-killed *M. vaccae* NCTC 11659 (SRL 172; batch A4, containing 10 mg wet-weight of bacilli per mL of M/l5 BBS at pH 8.0, equivalent to 10^9^ bacilli per dose; injected volume: 0.1 mL) together with standard immunotherapy for TB (isoniazid, also known as isonicotinic acid hydrazide (INH), rifampicin, and streptomycin for two months, followed by four months of INH and rifampicin alone). After one month, serum levels of IL-4, IL-10, and TNF decreased (*p* < 0.00l, *p* < 0.0l, and *p* < 0.01, respectively) while levels of IFNγ (*p* = 0.005) increased more in *M. vaccae* NCTC 11659-treated individuals than in those receiving drug therapy alone. Another randomized, placebo-controlled clinical trial conducted in Uganda included 120 HIV-negative adults with newly diagnosed pulmonary TB, recruited from August 1995 to February 1997 [56]. After screening, standard immunotherapy for TB began (two months of self-administered daily INH, rifampicin, pyrazinamide, and ethambutol, followed by four months of daily INH and rifampicin with doses adjusted for body weight). In addition to drug therapy, individuals received either a single intradermal injection of 0.1 mL heat-killed *M. vaccae* NCTC 11659 (containing 10^9^ organisms) in sterile BBS, or 0.1 mL sterile BBS placebo-excipient on the eighth day of anti-TB drug therapy. Heat-killed *M. vaccae* NCTC 11659 was generally safe and well tolerated. The major finding of the study was that the number of individuals receiving *M. vaccae* NCTC 11659 and having negative sputum cultures after one month of anti-TB treatment was significantly higher than the number of those with sputum culture conversion in the placebo group (35% in the *M. vaccae* NCTC 11659 group vs. 14% in the placebo group; *p* = 0.01). The results from the above-mentioned studies suggest that co-administration of *M. vaccae* NCTC 11659 favors a switch from Th2 to Thl immune response during *M. tuberculosis* infection, and this is associated with faster recovery and clinical benefits such as reduced recovery time from fever, improved smear conversion, and greater reduction in erythrocyte sedimentation rate (ESR). These results are in accordance with other studies conducted in the 1990s employing *M. vaccae* NCTC 11659 as an immunomodulatory agent in the treatment of TB in Nigeria [57] and Romania [54,154].

##### Repeated Intradermal *M. vaccae* NCTC 11659 Administrations as an Adjunct Therapy for First-Line Drug Therapy for Treatment of TB

In a follow-up study, Dlugovitzky and colleagues [41] administered Argentinian individuals with newly diagnosed pulmonary TB between 18–70 years of age with a triple-dose immunotherapy with heat-killed *M. vaccae* NCTC 11659 (SRL 172) combined with drug therapy for TB, consisting of daily INH, rifampicin, ethambutol, and pyrazinamide for two months followed by daily INH and rifampicin for a continuation phase of four months. *M. vaccae* NCTC 11659 was administered at days 1, 30, and 60 of drug therapy (10 mg of heat-killed *M. vaccae* NCTC 11659 suspended in 1.0 mL of BBS (pH 8); placebo contained BBS alone; a volume of 0.1 mL of *M. vaccae* NCTC 11659 (equivalent to 10^9^ bacilli per dose) or placebo was given by intradermal injection over alternating deltoid muscles). In confirmation of their previous study applying a single injection of *M. vaccae* NCTC 11659 [55], individuals receiving *M. vaccae* NCTC 11659 repeatedly showed faster clearance of tuberculous bacilli from sputum (*p* < 0.03), better radiological clearance of pulmonary cavities, and a faster fall in erythrocyte sedimentation rate (ESR; 63% vs. 35%; *p* < 0.001) compared to placebo-treated individuals. Serum TNF (*p* < 0.001) and IL-4 (*p* < 0.001) were lower in the group receiving *M. vaccae* NCTC 11659 vs. placebo.

##### Repeated Oral *M. vaccae* NCTC 11659 Administrations Promote Treatment of TB

A few years later, the same group conducted another clinical study to investigate whether *M. vaccae* NCTC 11659 (SLR 172) has beneficial effects in 10 individuals aged 16–52 with moderate to advanced pulmonary TB at Carrasco Hospital, Argentina, when administered via the non-invasive oral route [58]. All ten participants received two months of daily rifampicin, INH, ethambutol, and pyrazinamide followed by four months of daily rifampicin and INH. *M. vaccae* NCTC 11659 was absorbed into a gelatin made from potato starch/lactose powder (46 g starch to 184 g lactose) and encapsulated so that each gelatin capsule contained 1 mg of bacilli (10^9^). Each patient swallowed a single capsule on the first day of drug therapy, then on days 7, 14, 21, and 28. Thereafter, the capsules were taken at two fortnightly intervals, followed by monthly doses to the end of six months, for a total of ten doses. The results of this study indicated that *M. vaccae* NCTC 11659 is as successful when administered via the oral route as when given via intradermal injection in the treatment of TB, as shown by the negative sputum conversion, normalization of the ESR, recovery of body weight, increased IFNγ and IL-10 levels as well as decreased TNF levels from in vitro-cultured peripheral blood mononuclear cells (PBMCs), respectively) in *M. vaccae* NCTC 11659-treated TB patients, suggesting that *M. vaccae* NCTC 11659 can also induce its immunomodulatory effects via the mucosal immune system, where microfold cells (M cells) [155] phagocytize mycobacteria and mycobacterial antigens and transport them to macrophages in the epithelium [156].

More recently, a phase III clinical trial was conducted between 2014 and 2018, comprised of an ethnically diverse population of Ukrainian and Mongolian TB patients [43]. In this study, *M. vaccae* NCTC 11659 (V7, a hydrolyzed form of *M. vaccae* NCTC 11659) was administered in the form of an oral tablet containing 10 µg of hydrolyzed and heat-killed bacteria, administered once-daily for one month, in combination with standard TB drug therapy consisting of daily doses of INH (300 mg), rifampicin (600 mg), ethambutol (1200 mg), pyrazinamide (2000 mg), and streptomycin (1000 mg). The results of this phase III study indicate that when daily oral administrations of *M. vaccae* NCTC 11659 are combined with TB drug therapy, the *M. tuberculosis* clearance rate in the sputum is significantly improved compared to the placebo group receiving TB drug therapy only, as is body weight gain (eight fold higher than placebo) and reduction in ESR (72% in the *M. vaccae* NCTC 11659-treated group vs. 53.8% in the placebo-treated group). These results support the findings of two prior phase II trials comprising individuals with diverse forms of TB and using two different mycobacteria (i.e., *M. vaccae* NCTC 11659 [44] and *M. vaccae* ATCC 15483^T^ (Longcom batch; No M20111124)) [51] administered with the same dose and formulation. Of note, in the here referenced clinical trials [43,44,51], *M. vaccae* NCTC 11659 and *M. vaccae* ATCC 15483^T^ (Longcom batch) were administered daily for one month at a dose of 10 µg (equivalent to 10^7^ bacilli per dose), which is 100-fold lower than the oral dose administered earlier by Dlugovitzky and colleagues in a weekly, two-weekly, or monthly fashion (ten doses of 1 mg each) [58].

##### Repeated Intradermal *M. vaccae* NCTC 11659 Administration Prevents TB in Persons with HIV Infection

HIV infection is a major contributor to the TB epidemic, and neither INH preventive therapy (IPT) nor antiretroviral therapy (ART) is completely effective in reducing the infection risk [157,158,159,160]. Consequently, TB remains the major cause of death in most regions where TB and HIV coexist [157,158,161] and represents the most important opportunistic infection affecting HIV-positive people in the developing world [42,142]. Therefore, von Reyn and colleagues [42] conducted a randomized, placebo-controlled, double-blind clinical trial (DarDar trial) in Tanzania investigating the hypothesis that mycobacterial immunity primed by childhood BCG immunization has to be boosted by mycobacterial re-exposure to provide protection against TB in patients with HIV infection. The authors further hypothesized that a successful prime-boost strategy against TB in HIV infection would need to meet the following criteria: (1) given early in HIV infection for an optimal immune response; (2) present multiple antigens because of the reduced T cell diversity in HIV infection; and (3) be well tolerated and, therefore, excluding the possibility of administering live mycobacteria. As timely administration of an inactivated whole-cell mycobacterial reagent would fulfill all these criteria, the authors employed the same *M. vaccae* NCTC 11659 formulation developed by Stanford and Rook (Strain R877R NCTC 11659, 10^9^ bacilli in 0.1 mL) [49]. Although single-dose studies [56,162] turned out to be unsuccessful, a phase II study [163] in Zambia indicated that five doses of heat-killed *M. vaccae* NCTC 11659 administered intradermally promoted mycobacteria-directed T cell responses in HIV-infected participants and that responses were maximal in recipients primed with BCG during childhood. Of note, studies conducted beforehand have demonstrated the safety of a multiple-dose series of intradermal *M. vaccae* NCTC 11659 in healthy adults and in HIV-infected adults and children [47,164,165]. In line with these findings, a subsequent, randomized, controlled, phase II trial [48] in Finland demonstrated that five doses of intradermal *M. vaccae* NCTC 11659 (MV 007) were well-tolerated in HIV-infected participants and boosted mycobacteria-directed T cell responses in recipients primed with BCG. The above-mentioned DarDar trial [42] aimed at determining whether repeated administrations of inactivated whole cell *M. vaccae* NCTC 11659 could boost childhood BCG vaccination to increase protection against TB and whether it could also prove successful in preventing HIV-associated TB among BCG-primed recipients in a TB endemic country such as Tanzania [45]. To be eligible for the study [42], participants had to be HIV-positive, at least 18 years of age, with a CD4 T cell count of at least 200 cells/mL, a visible BCG scar from childhood immunization (sensitivity > 90%, as reported in a study conducted in the Malawi region [166]), a negative pregnancy test, and no evidence of active TB. A total of 2013 individuals entered the study, were randomized (1006 to *M. vaccae* NCTC 11659 and 1007 to placebo), and followed [42]. Individuals in the *M. vaccae* NCTC 11659 group received a five-dose series of 0.1 mL intradermal *M. vaccae* NCTC 11659 (SRL 172, 1 mg, 10^9^ colony-forming units in BBS; Immodulon, London, UK), while those in the placebo group received BBS (same appearance, identical vial) at respective time points over the deltoid at 0, 2, 4, 6, and 12 months. Tuberculin skin tests were performed every three months for a median of 3.3 years, and individuals with reactions of at least 5 mm were administered INH for six months. Blood sampling was performed at baseline (prior to administering either BBS or *M. vaccae* NCTC 11659) and two months after the final (fifth) dose of treatment, in order to assess PBMC proliferation and IFNγ production and serum IgG against lipoarabinomannan, a widely expressed mycobacterial lipopeptide [167]. Other outcome measures were “disseminated (primary endpoint)”, “definite”, and “probable TB (secondary endpoints)”. In confirmation with the phase II study results, this phase III study demonstrated that a multiple-dose series of inactivated *M. vaccae* NCTC 11659 given to BCG-primed recipients with HIV infection in Tanzania significantly (39%) reduced the risk of developing HIV-associated definite TB [42]. Overall, repeated immunizations were well-tolerated, with no adverse effect on CD4^+^ T cell counts or HIV viral load, and no increase in the rate of serious adverse events was recorded. Noteworthy, another study showed that PBMCs isolated from HIV-infected and BCG-vaccinated adults with a CD4^+^ count ≥ 200 cells/mL administered with five intradermal doses of whole cell heat-inactivated *M. vaccae* NCTC 11659 further showed a boosted IFNγ production and proliferation when exposed in vitro to *M. vaccae* NCTC 11659 sonicated at a concentration of 2 µg/mL over five days [45]; in addition, an increased serum antibody response to lipoarabinomannan, indicative of protective immunity against TB, was detected following treatment with *M. vaccae* NCTC 11659 in HIV-infected adults [45]. More recently, the same authors showed similar BCG-boosting effects of *M. vaccae* NCTC 11659 in mice [168]. Briefly, mice were vaccinated with BCG (TICE strain, 1 × 10^5^ colony forming units (CFU) in saline, intradermal (i.d.), week 0), administered with two doses of *M. vaccae* NCTC 11659 (DAR-901; SRL 172; i.d., 1 mg/50 µL citrate buffer/dose on weeks 12 and 14) and infected with aerosolized *M. tuberculosis* (H37Rv strain, 100 CFU/lung/mouse, week 20). The results show that two doses of DAR-901 (equivalent to 10^9^ bacilli per dose) can boost the efficacy of BGC vaccine, as shown by a significant reduction in the number of *M. tuberculosis* cells from lungs and spleen of infected mice in the group receiving BCG + two doses of *M. vaccae* NCTC 11659 compared to the group receiving BCG alone. In addition, this effect was paralleled by increased IFNγ secretion in splenocytes of *M. vaccae* NCTC 11659-treated mice [168].

The above-mentioned clinical studies are consistent with the first observational studies in mice [119,169,170] showing that *M. vaccae* NCTC 11659 suppresses Th2 while boosting Th1 immune response in the host, resulting in significantly increased protection against, and clearance of, *M. tuberculosis* bacilli.

#### 2.1.2. *M. vaccae* NCTC 11659 and Leprosy

Leprosy is a chronic granulomatous infection caused by the obligate intracellular organism *M. leprae*, which primarily affects the skin and peripheral nerves [171,172] with a peculiar affinity for Schwann cells, resulting in demyelination and loss of axonal conductance of peripheral nerves [173]. There are two major types of clinical leprosy. Tuberculoid leprosy is characterized by a vigorous cellular Th1 immune response to the bacterium, which limits the disease to a few well-defined skin patches or nerve trunks [174]. These are infiltrated by IFNγ, TNF, IL-12, IL-15, and IL-18-secreting CD4^+^ T lymphocytes [175,176,177,178] forming granulomas containing multinucleated giant cells that prevent the bacterium from spreading [175]. In contrast, lepromatous leprosy lacks a specific cellular immune response and lesions are rich in cells secreting the Th2 cytokines IL-4 and IL-10 [175], allowing uncontrolled proliferation of leprosy bacilli with many lesions and extensive infiltration of the skin and nerves [173]. Most individuals have intermediate forms, which are clinically unstable and can shift toward either the tuberculoid or the lepromatous pole. Thus, these findings from persons infected with *M. leprae* suggest, similarly to what is the case for *M. tuberculosis*, that a Th1 rather than Th2 immune response is beneficial in containing the disease. As previous studies have shown that *M. vaccae* NCTC 11659 modulates the immune response via Th1 polarization [51,103,107], and as this immunomodulatory approach has been shown to be effective in the prevention and treatment of TB [55,58,152,179], studies were conducted in humans investigating whether the immunomodulatory properties of *M. vaccae* NCTC 11659 would also be beneficial for people with leprosy. The first clinical trials in humans started in Spain between 1983–1985 amongst volunteers with long-treated lepromatous leprosy to determine the dose of *M. vaccae* NCTC 11659 required to induce a positive skin test response to leprosin A [62]. The latter was originally isolated from *M. leprae* bacilli extracted from the tissues of experimentally infected armadillos. The rationale behind the use of the leprosin A skin test in this study was that individuals with lepromatous leprosy do not respond to leprosin A, whereas individuals with tuberculoid leprosy show a positive response [180]. Thus, a positive skin response to leprosin A in individuals with lepromatous leprosy would indicate a favorable shift toward a less dangerous tuberculoid type of leprosy. Moreover, studies conducted in India [181] and Malawi [182] have shown that skin test positivity to leprosin A correlates with protection from subsequent development of lepromatous leprosy. In the study from Stanford and colleagues [62], leprosin A negative individuals with lepromatous leprosy were treated i.d. at yearly intervals with ascending doses of *M. vaccae* NCTC 11659 (10^7^, 10^8^, 10^9^, equivalent to 0.01, 0.1 and 1 mg wet weight of *M. vaccae* NCTC 11659, respectively) or BBS. Interestingly, one year after the 10^9^ dose, about one third of participants developed positive responses to leprosin A for the first time, suggesting that a shift from Th2 to Th1 cellular response against *M. leprae* requires at least a 1 mg dose of *M. vaccae* NCTC 11659 and several months to develop. Another study showed that i.d. administration of *M. vaccae* NCTC 11659 (one single injection of 10^8^ heat-killed bacilli in 0.1 mL BBS) to healthy individuals with regular contact with people with leprosy increased immune responses against leprosin A, suggesting the use of *M. vaccae* NCTC 11659 as a potential vaccine against lepromatous leprosy [59]. Of note, so far and similarly to what has been shown for TB, only vaccination using BCG is considered to be effective in reducing the risk for developing leprosy [183,184]. Thus, studies were conducted to establish whether *M. vaccae* NCTC 11659 could also induce protective immunity against leprosy, or boost the efficacy of BCG vaccination. In a study by Truoc and colleagues [60] performed in Vietnam, children living in close contact with persons with leprosy were vaccinated with a single i.d. injection of BCG alone, BCG + 10^8^ heat-killed *M. vaccae* NCTC 11659 (R877R), or 10^9^ heat-killed *M. vaccae* NCTC 11659 (R877R) alone. The results showed that although all three vaccines significantly increased the number of recipients being skin-test positive to leprosin A, the best protection was seen in children receiving BCG + *M. vaccae* NCTC 11659. Similar protective effects of *M. vaccae* NCTC 11659 against leprosy were also found in other studies carried out in India [185], Iran [186,187,188], and Lebanon [189]. Another study showed that immunotherapy with *M. vaccae* NCTC 11659 (i.d., 1 mg wet weight in 0.1 mL BBS) increased skin capillary blood flow, important for a proper transport of oxygen and nutrients [190,191] and increased skin temperature, a marker for leprosy severity (cooler tissues are more severely affected [192]), in individuals with chronic leprosy [61].

#### 2.1.3. *M. vaccae* NCTC 11659 and Psoriasis

Psoriasis is a chronic autoimmune skin disease characterized by the production of erythematous squamous lesions with abnormal keratinocyte proliferation, vascular alterations, and dermal–epidermal inflammatory infiltrates [193], with plaque psoriasis being the most common variant [194]. Although the pathogenesis of this disease remains poorly understood, lesions are likely to be mediated by activated Th cells releasing growth promoting and pro-inflammatory cytokines [195,196]. Interest in the use of *M. vaccae* NCTC 11659 for treatment of psoriasis started with the observation that *M. vaccae* NCTC 11659 ameliorated psoriasis in persons with and without comorbid leprosy [197]. In a clinical study conducted later by Lehrer and colleagues [63], individuals with chronic plaque psoriasis were recruited. To clinically assess psoriasis, an index taking into account the extent of the affected skin and the intensity of erythema, desquamation, and infiltration was used (psoriasis area severity index (PASI) [198]). Participants received a single dose of *M. vaccae* NCTC 11659 (Batch A4, 1 mg, i.d.) or placebo (tetanus toxoid, Tetavax: Merieux, Institut Pasteur). As a result, the recipients of *M. vaccae* NCTC 11659 showed a reduced PASI, indicating improved skin lesions, and reduced blood lymphoproliferative response to concanavalin A when compared with placebo recipients six months after treatment. Of note, tetanus toxoid was chosen as a placebo because it produces a small local response, helping to maintain study blindness and had some benefit for the participants. Although the underlying mechanisms are not fully understood, the authors of the study suggested that a reduction in the toxic effects of TNF, known to be high in psoriasis skin lesions [199,200], plays an important role. This hypothesis is also supported by other studies suggesting TNF to have higher toxicity when Th2 cytokines prevail over Th1 [140] and that the Th1-polarizing effect of *M. vaccae* NCTC 11659 overall contributes to the clinical improvement in TB patients [58].

#### 2.1.4. *M. vaccae* NCTC 11659 and Atopic Dermatitis

Atopic dermatitis is an inflammatory skin disorder characterized by intense itching and recurrent eczematous lesions with usual age of onset in early childhood [201,202]. The prevalence of atopic dermatitis has doubled during the last half century in Western society [203] and this has been attributed to a reduced microbial exposure including to infectious diseases such as TB [204,205,206]. Supporting the role of reduced exposure to TB in the increasing incidence rates of atopic dermatitis, it was shown that reduced exposure to mycobacteria is associated with an increased prevalence of atopic dermatitis and asthma [207]. Based on these observations, Arkwright and David hypothesized in their clinical trial that immunizing atopic individuals with *M. vaccae* NCTC 11659 ameliorates their disease [64]. This hypothesis is further supported by animal data showing that *M. vaccae* NCTC 11659 induces anti-inflammatory responses in animal models of atopy [70]. In their study, Arkwright and David immunized children aged 5–18 years with moderate-to-severe atopic dermatitis with an i.d. injection of 0.3 mL of a heat-killed preparation of *M. vaccae* NCTC 11659 (SRL 172, 10^10^ organisms per mL). The severity of the children’s dermatitis was assessed just before treatment and then at one and three months after treatment with a score accounting for erythema, excoriation, exudation, and lichenification [208]. Serum total IgE concentration as well as absolute blood eosinophil counts were measured at the same time points. The results of this study showed that children with atopic dermatitis receiving *M. vaccae* NCTC 11659, relative to children receiving BBS, showed reduced surface area of dermatitis lesions at one month as well as three months after treatment, although no child showed a complete resolution of the disease. On the other hand, no significant reduction in the amount of serum IgE or in the absolute eosinophil count were measured. Although the cellular mechanisms by which *M. vaccae* NCTC 11659 exerted these effects were not the focus of the study, the authors hypothesize a prominent role of Langerhans cells. These cells are the major antigen-presenting cells in the skin and, compared to other types of DCs, express large amounts of the non-classical major histocompatibility complex (MHC) receptor CD1a on their cell surface, which is known to present lipid antigens, especially those derived from mycobacteria [209]. Moreover, there is evidence that IL-10 and transforming growth factor beta 1 (TGFβ1) are important mediators in inducing tolerance and preventing atopy [210], and it was shown that polymorphisms of the *TGFB1* gene associated with low production of this cytokine predisposes individuals to atopic dermatitis [67]. Thus, it is likely that *M. vaccae* NCTC 11659 may restore adequate levels of those cytokines, a hypothesis that is also supported by studies in mice showing increased numbers of Tregs secreting IL-10 and TGFβ1 in a model of allergy [40]. Noteworthy, younger children aged 2–6 years with atopic dermatitis did not benefit from a subdermal single dose of *M. vaccae* NCTC 11659 (SRP299; 1 mg in 0.1 mL) with respect to the surface area of dermatitis lesions [38]. This was also confirmed in a large cohort study [37] involving male and female atopic children (aged five to 16 years) of 19 different centers between the United Kingdom and Croatia with dermatitis severity required to be moderate to severe according to the six area, six sign, atopic dermatitis score [208]. Participants in this study [37] received either *M. vaccae* NCTC 11659 (SRP 299; 1 or 0.1 mg) or placebo (PBS) as a single 0.1 mL i.d. injection.

#### 2.1.5. *M. vaccae* NCTC 11659 and Asthma

Bronchial asthma is a chronic airway inflammatory disease characterized by a predominant Th2 over Th1 immune response [211] with large production of IL-4 and IL-5, which in turn promote airway eosinophilia and IgE synthesis [212]. Interestingly, the prevalence of asthma is higher in developed, Westernized countries and relatively low in developing countries [213]. Moreover, migration studies indicate that immigrants from countries with a lower asthma incidence than the natives of the host country show rising incidence rates with increasing length of residence, further suggesting that environmental factors play an important role in the etiopathogenesis of the disease [214,215]. Epidemiological studies explain environment-dependent differences in asthma incidence with the “hygiene hypothesis”, according to which the increased prevalence of atopic diseases has been, at least partly, due to reduced early childhood exposure to environmental microbes, resulting in inadequate development of immunity against infectious agents and inappropriate inflammation in response to harmless antigens [216,217,218,219]. *M. vaccae* NCTC 11659 is known to shift the immune response from Th2 to Th1 [4,103] and studies involving animal models of allergic asthma revealed both a suppressive effect of *M. vaccae* NCTC 11659 on IL-5 and IgE synthesis in ovalbumin (OVA)-sensitized mice [70] as well as an inductive effect on Tregs, which in turn downregulated Th2 responses [40]. Therefore, a placebo-controlled phase I clinical trial was designed to test the hypothesis that *M. vaccae* NCTC 11659 is protective in asthmatic humans [65]. In this clinical trial, a total of 24 asthmatic male volunteers with mild-to-moderate disease received a single i.d. injection of *M. vaccae* NCTC 11659 (SRL 172, 1 mg in 0.1 mL BBS, equivalent to 10^9^ bacilli per dose) or BBS alone on day 0 of the experimental protocol. A bronchial allergen challenge [220] was performed on days −14 and 21. PBMCs were isolated right before *M. vaccae* NCTC 11659 injection on day 0 and subsequently on days 21 and 42, and cultured for 48 h with or without dust mite allergen extract from *Dermatophagoides pteronyssinus*. In support of an asthma-protective effect of *M. vaccae* NCTC 11659, participants receiving the latter showed a by trend (*p* = 0.06) decreased area under the curve (AUC) for IL-5 concentration in the culture supernatants considering the three time points investigated when compared with participants receiving the placebo. As a similar trend was also seen in serum IgE (*p* = 0.07), these data suggest that *M. vaccae* NCTC 11659 potentially has beneficial effects in asthmatic patients.

#### 2.1.6. *M. vaccae* NCTC 11659 and Cancer

Interest in the use of mycobacteria in the treatment of cancer started about a hundred years ago, when the first studies indicated lower cancer risk in TB patients [221,222]. As a consequence, anti-tumor effects of BCG vaccine were tested [223], and proved to be successful against bladder carcinoma [224,225]. Moreover, BCG reduces the susceptibility to develop malignant melanoma and increases successful treatment outcome, both by about 50% [226]. Of note, contrary to *M. vaccae* NCTC 11659, BCG does not modulate from Th2 toward Th1 maturation of T cells, which is an essential step in effective immunotherapy against cancer [103,223]. Therefore, the mechanisms through which BCG influences the development of certain cancers remains largely unknown. Studies were performed in which persons with a variety of different malignancies received repeated i.d. injections of *M. vaccae* NCTC 11659 (SRL 172, 1 mg in 0.1 mL BBS), resulting in a significant improvement in quality of life scores [68], better tolerance of drug therapy side effects [94], and increased survival [95]. Other studies confirmed cancer protective effects of *M. vaccae* NCTC 11659 (SRL 172), indicated, for instance, by an improved survival of participants with melanoma [66,67] as well as advanced prostate cancer—the latter was paralleled by a switch from Th2 to Th1 polarization [137]. O’Brien and colleagues performed a randomized phase II trial in which *M. vaccae* NCTC 11659 (SRL 172) was administered once a week for three weeks and then once a month for three to six months via i.d. injections (10^9^ bacilli; 1 mg in 0.1 mL BBS) together with intravenous injection of drug therapy in individuals with inoperable non-small-cell lung adenocarcinoma and mesothelioma. The combination of drug therapy with *M. vaccae* NCTC 11659 immunotherapy improved the participants’ median and one year survival, sleep, and appetite but did not affect the serum Th1 cytokines IFNγ and TNF [93]. Although a beneficial interaction between drug therapy and *M. vaccae* NCTC 11659 administration was confirmed in small cell lung cancer patients two years later [227], a follow-up phase III study from O’Brien and colleagues in 2004 revealed that five i.d. injections of *M. vaccae* NCTC 11659 (SRL 172, 1 mg in 0.1 mL BBS) once a month following standard drug therapy only by trend prolonged the survival of participants with advanced non-small-cell lung adenocarcinoma. Interestingly, the latter trial found that *M. vaccae* NCTC 11659 co-administration significantly improved the participants’ cognitive functioning and vitality while reducing treatment-related adverse effects such as nausea, vomiting, peripheral neuropathy, body pain, and dyspnea [68]. Of note, re-analysis of the data revealed that participants with better compliance also showed a strongly increased survival rate [95], suggesting that the lack of a significant effect for cancer protection in the initial study [68] could be explained by the poor compliance of study participants. In line with cancer-protective effects of *M. vaccae* NCTC 11659, a phase II clinical trial further showed promising effects of a related species, *M. obuense* NCTC 13365 (IMM-101, six i.d. injections of 1 mg in 0.1 mL BBS; 3 doses administered every two weeks, followed by four weeks rest; the remaining three doses were administered every four weeks) in advanced pancreatic ductal adenocarcinoma [228].

### 2.2. Mechanistic Studies on the Protective Effects of M. vaccae NCTC 11659 and M. vaccae ATCC 15483^T^

**Figure 1:**
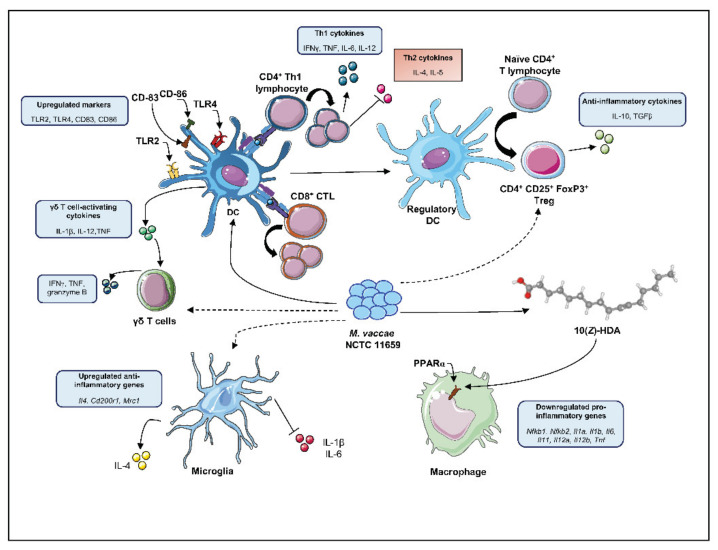
Pleiotropic effects of *M. vaccae* National Collection of Type Cultures (NCTC) 11659 on the immune system, promoting immunoregulation. Recognition of *M. vaccae* NCTC 11659 by dendritic cells (DCs) results in the upregulation of Toll-like receptor (TLR)2, TLR4, and of the maturation markers cluster of differentiation (CD)83 and CD86. It also results in the polarization and proliferation of CD4+ T lymphocytes toward a T helper (Th)1 phenotype with production of Th1 cytokines like interferon gamma (IFNγ), tumor necrosis factor (TNF), interleukin (IL)6, and IL-12. *M. vaccae* NCTC 11659-stimulated DCs also promote the differentiation of CD8+ cytotoxic T lymphocytes (CTL) and gammadelta (γδ) T cells with antitumor activity as well as of CD4+CD25+ forkhead box P3 (FoxP3)^+^ regulatory T cells (Treg). *M. vaccae* NCTC 11659 upregulates anti-inflammatory genes (i.e., *Il4*, *Cd220r1*, *mannose receptor C-type 1* (*Mrc1*)) in hippocampal microglia, indicated by increased secretion of IL-4, while in contrast reducing secretion of proinflammatory markers such as IL-1β and IL-6. Finally, the *M. vaccae* NCTC 11659-produced lipid 10(*Z*)-hexadecenoic acid (10(*Z*)-HDA) induces anti-inflammatory responses in isolated peritoneal macrophages via activation of peroxisome proliferator-activated receptor alpha (PPARα) and downregulation of proinflammatory genes (i.e., *transcription factor nuclear factor-kappa B* (*Nfkb1, Nfkb2*), *Il1a*, *Il1b*, *Il6*, *Il11*, *Il12a*, *Il12b*, *Tnf*). Solid-line arrows represent direct effects of *M. vaccae* NCTC 11659 while dashed-line arrows represent indirect effects. 3D image of 10(*Z*)-HDA retrieved from pubchem.ncbi.nlm.nih.gov.

As extensively reported above, one of the most acknowledged mechanisms through which *M. vaccae* NCTC 11659 exerts its immunomodulatory effects is by facilitating the development of naïve T cells into Th1 instead of Th2 cells, and this proved to be beneficial in the above-discussed pathologies characterized by an imbalanced Th2 over Th1 immune response. However, the mechanisms of action of *M. vaccae* NCTC 11659 seem to be much more complex than that. Therefore, in the following sections, studies unraveling further mechanistic details about the immunomodulatory effects of *M. vaccae* NCTC 11659 are discussed and summarized in Figure 1. Studies investigating the mechanisms of action of the closely related strain, *M. vaccae* ATCC 15483^T^, are also presented. In detail, besides the impact of both bacterial species on the Th1/Th2 immune profile, their effects on DCs, CD11b^+^ myeloid cells, γδ T cells, CD8^+^ CTL, and Tregs are also outlined. As measures of absolute and relative immune cell numbers, i.e., neutrophils and monocytes, are particularly emerging as important predictors of anxiety-disorders, affective disorders, trauma and stressor-related disorders, and suicide, it is hoped that understanding the impact of *M. vaccae* NCTC 11659 on peripheral immune function may inform potential mechanisms through which *M. vaccae* NCTC 11659 promotes stress resilience. For example, inflammation in general is thought to increase the risk of anxiety disorders [229], affective disorders [230,231], trauma and stressor-related disorders including PTSD [232], and suicide [233]. In support, increases in granulocyte:lymphocyte ratios, neutrophil cell counts, or neutrophil:lymphocyte ratios, thought to be a reliable marker of chronic low-grade inflammation [234,235], have been associated with increases in blood inflammatory markers, major depressive disorder (MDD) [236,237], impulsivity [238], and suicidal behavior [239,240,241,242]. In addition, an increase in the number of circulating monocytes is also thought to reflect chronic inflammation, and has been identified, among the white blood cell subtype counts, to be an independent predictor of cardiovascular disease risk [243]. Recently, in a study of polygenic, epigenetic, metabolomic, endocrine, inflammatory, and routine clinical lab markers, computerized neurocognitive testing, and symptom self-reports, machine learning models revealed that absolute numbers of monocytes measured prior to deployment of soldiers to Afghanistan were among the highest ranking predictors of provisional PTSD diagnosis 90–180 days post-deployment [232]. Finally, monocyte:lymphocyte ratios have also been shown to be predictive of a chronic inflammatory state [244], and the inflammatory state of monocytes has been linked to depression severity, childhood adversity, and suicide risk [230,245,246,247]. Given preclinical studies suggesting that stress-mobilized IL-6-secreting inflammatory monocytes from the bone marrow traffic to the brain and mediate stress-induced anxiety- and depressive-like behavioral responses [248,249,250,251,252,253], understanding the effects of *M. vaccae* NCTC 11659 on peripheral immune signaling may inform potential mechanisms through which *M. vaccae* NCTC 11659 promotes stress resilience.

#### 2.2.1. *M. vaccae* NCTC 11659 Effects on DCs and Th1/Th2 Immune Profile

In their in vitro study, Le Bert and colleagues [46] investigated the effects of *M. vaccae* NCTC 11659 on human DC maturation. PBMCs from healthy participants were used to isolate CD14^+^ monocytes, which were subsequently differentiated into DCs during a 4–day incubation with granulocyte-macrophage colony-stimulating factor (GM-CSF) and IL-4. DCs were then cultured for 24 h in the presence of different doses of heat-killed *M. vaccae* NCTC 11659 (batch MV07, 1 µg/mL, 10 µg/mL, 100 µg/mL), before they were co-cultured with naïve CD4^+^ T cells. While *M. vaccae* NCTC 11659 in a dose-dependent manner promoted maturation of DCs, indicated by upregulation of the co-stimulatory molecule CD86 and the maturation marker CD83 [254], co-culturing of these *M. vaccae* NCTC 11659-primed DCs with naïve T cells also reduced the number of IL-4^+^ T cells in a dose dependent manner, with the greatest reduction assessed at the 100 µg/mL dose. Together with the above referenced studies, these findings suggest that *M. vaccae* NCTC 11659 is mediating the shift from Th2 to Th1 immunity and, thus, its allergy protective effects [255,256], at least in part, via DCs. Of note, although this *M. vaccae* NCTC 11659-mediated inhibition of Th2 responses was critically dependent on TLR2 activation, and as other mycobacterial strains also activate TLR2 signaling [257,258,259], using a specific TLR2 ligand alone (i.e., Pam_3_CSK4; (Pam_3_CysSerLys4), a synthetic triacylated lipopeptide and a TLR2/TLR1 ligand) failed to induce a Th2 polarization of T cells, suggesting that TLR2 signaling alone is not sufficient to mediate this effect. Although further studies are certainly required to fully understand the underlying mechanisms, the inhibiting effect of *M. vaccae* NCTC 11659 on Th2 polarization of T cells was dependent on selective activation of the transcription factor cAMP-response element binding protein (CREB) in DCs, which can antagonize the pro-inflammatory transcription factor nuclear factor-kappa B (NF-κB) and upregulate IL-10 [260]. Interestingly, mycobacteria have also been shown to induce the CREB signaling pathway in macrophages [261,262] and PBMCs [263]. Evidence for *M. vaccae* NCTC 11659 to promote a Th1 over Th2 polarization comes from mouse models of OVA-induced asthma. In an early work from Wang and Rook [70], mice were sensitized with OVA (on day 0 and 21) and then a single s.c. dose of *M. vaccae* NCTC 11659 (10^7^, 10^8^, or 10^9^ bacilli in saline solution) was administered 42 days after the first OVA challenge. *M. vaccae* NCTC 11659, irrespective of the concentration administered, significantly lowered the levels of serum IgE compared to vehicle (Veh)-treated mice. Furthermore, splenocytes cultured in vitro from *M. vaccae* NCTC 11659-treated mice showed decreased levels of the Th2 cytokine IL-4 and increased levels of the Th1 cytokine IL-2 in the supernatants. Similar results were also obtained by Smit and colleagues [69], who administered *M. vaccae* NCTC 11659 (SRL 172; 10^6^, 10^7^, or 10^8^ CFU in 0.1 mL BBS injected s.c.) immediately before challenging mice with OVA, although only the 10^7^ dose of *M. vaccae* NCTC 11659 effectively prevented OVA-induced increases in eosinophil count and increases in IL-4 and IL-5 in the BAL fluid. In addition, *M. vaccae* NCTC 11659 significantly reduced the levels of IgE and IgG1 antibodies in the serum of mice after OVA challenge.

In support also of *M. vaccae* NCTC 11659-related strains affecting DC function, a study by Strygin and colleagues [264] found effects of both heat-killed and sonicated *M. vaccae* ATCC 15483^T^ (DSM 43292^T^) on DC function in co-cultures of DCs and CD4^+^ T cells from both human and murine sources. In their study, *M. vaccae* ATCC 15483^T^ was cultured in Middelbrook 7H9 medium, 10^10^ cells/mL were diluted in PBS, and either sonicated on ice or autoclaved for 15 min at 120 °C. Mouse DCs were differentiated from bone marrow cells following erythrocyte lysis (eight days of culture in the presence of 100 ng/mL GM-CSF). DCs were stimulated with 10 µg/mL of heat-killed or lysate *M. vaccae* ATCC 15483^T^ (3.2 x 10^5^ DCs/mL, 24 h) and 10^4^ DCs were co-incubated with 10^5^ spleen-derived naïve allogeneic CD4^+^CD62L^+^ T lymphocytes for five days. Human DCs and CD4^+^ T lymphocytes were isolated from PBMCs of healthy donors using magnetic separation (CD4, CD14 microbeads). Isolated cells were cultured in the presence of 100 ng/mL GM-CSF and 50 ng/mL IL-4 with and without heat-killed/sonicated *M. vaccae* ATCC 15483^T^ for four days. Considering both human and murine DCs, heat-killed and sonicated *M. vaccae* ATCC 15483^T^ both induced the upregulation of the co-stimulatory marker CD86 and of the maturation marker CD83, although sonicated *M. vaccae* ATCC 15483^T^ increased the expression of CD86 to a greater extent compared to heat-killed *M. vaccae* ATCC 15483^T^. The secretion of TNF and IL-10 was also significantly increased in the supernatants of lysate vs. heat-killed *M. vaccae* ATCC 15483^T^ co-cultures, while no differences were detected between the two bacterial preparations in terms of the supernatant cytokines IL-6, IL-12p70, IL-1β, IL-4, IL-5, IL-13, IFNγ, and IL-17.

#### 2.2.2. *M. vaccae* NCTC 11659 Effects on γδ T Cells

The γδ T cells are characterized by the expression of the γ and δ chain in their TCR (T cell receptor) [265] and NKG2D (natural killer group 2D) [266]. They have cytotoxic activity and show protective immunosurveillance against cancerous cells [267,268], being able to recognize antigens independent of MHC I, which is often downregulated in a variety of cancers [269]. Interestingly, γδ T cells are highly reactive against mycobacterial antigens [270] and display vigorous cross-reactivity against tumor cells [271]. In order to investigate the mechanisms underlying the cancer protective effects of *M. vaccae* NCTC 11659 immunotherapy as reported above, Fowler and colleagues [71] focused on γδ T cells. In detail, following overnight stimulation of PBMCs isolated from healthy donors with heat-killed *M. vaccae* NCTC 11659 (100 µg/mL in BBS), the percentage of IFNγ and TNF secreting γδ T cells was increased. These Th1 cytokines have documented anti-tumor effects including upregulation of MHC class I molecules on the surface of tumor cells promoting recognition by cytotoxic CD8^+^ T cells [272,273], induction of cell cycle arrest and apoptosis in tumor cells [274,275], and facilitation of anti-tumor Th1 cell differentiation [276,277]. *M. vaccae* NCTC 11659 further upregulated the expression of granzyme B in γδ T cells, which is an important effector molecule in γδ T cell-induced cytotoxicity [278]. Interestingly, these effects are mediated by the direct activation of type 1 myeloid DCs, which in turn activate γδ T cells via production of IL-12, IL-1β, and TNF.

#### 2.2.3. *M. vaccae* NCTC 11659 Effects on CD11b^+^ Myeloid Cells

In order to gain more detailed insights into the modulatory effects of *M. vaccae* NCTC 11659 on the immune system, human PBMCs were isolated from healthy volunteers and incubated with *M. vaccae* NCTC 11659 (300 µg/mL) for 3 h [72]. Interestingly, the data revealed that the phagocytic cells, namely neutrophils and monocytes, are the main direct targets of the bacterium. Specifically, with respect to surface markers involved in adhesion/trafficking, co-culturing of PBMCs with *M. vaccae* NCTC 11659 downregulates CD62L and upregulates CD18, CD11a, CD44, CD54, and CD58k on monocytes. Among the co-stimulatory receptors and antigen presentation molecules, CD80, CD86, CD45, and CD137L were upregulated on monocytes following stimulation with *M. vaccae* NCTC 11659. Interestingly, a previous study suggests that increasing the expression of costimulatory receptors on monocytes facilitate anti-tumor immunity [91]. Finally, in vitro co-incubation of human PBMCs with *M. vaccae* NCTC 11659 further upregulates the PRRs TLR2, TLR4, CD14, CD36, and CD206. Therefore, although further functional studies are clearly required to fully understand the meaning of these changes, *M. vaccae* NCTC 11659 seems to have direct effects on CD11b^+^ neutrophil and macrophage function and, consequently, to affect first-line defense provided by the innate immune system as well as antigen presentation required for an adequate adaptive immunity [279]. Support for *M. vaccae* NCTC 11659 to affect the function of CD11b^+^ innate immune cells comes from the finding that stimulation of human whole blood cultures with *M. vaccae* NCTC 11659 for 24 h also resulted in increased production of the typical myeloid cell line-derived cytokines IL-6, IL-10, and TNF [72]. Interestingly, the secretion of signature T cell cytokines IFNγ, IL-2, and IL-4 by in vitro cultured human whole blood was not affected by *M. vaccae* NCTC 11659 co-incubation, at least not when stimulation was performed over 24 h, suggesting that *M. vaccae* NCTC 11659 specifically modulates the function and activity of CD11b^+^ myeloid cells of the innate immune system and, in an indirect process involving innate immune cells with antigen presenting function as well as the adaptive immune system and here specifically T cells [72].

Although the mechanisms through which *M. vaccae* NCTC 11659 specifically modulates the function and activity of CD11b^+^ myeloid cells of the innate immune system are not fully understood, recent studies have identified a mycobacteria-specific lipid, 1,2,3-tri [*Z*-10-hexadecenoyl] glycerol, isolated from *M. vaccae* NCTC 11659, that may play an important role [76]. Studies using RNA-Seq analysis of mRNA expression in freshly isolated murine peritoneal macrophages have shown that the free fatty acid form of the lipid, 10(*Z*)-HDA, suppresses lipopolysaccharide (LPS)-induced inflammation. Genes that were downregulated by 10(*Z*)-HDA included those encoding the transcription factor NF-κB (i.e., *Nfkb1, Nfkb2*), *Irf8*, pro-inflammatory cytokines (i.e., *Il1a*, *Il1b*, *Il6*, *Il11*, *Il12a*, *Il12b*, and *Tnf*), chemokine ligands, (i.e., *Ccl2*, *Ccl3*, *Ccl4*, *Ccl7*, *Ccl6*, *Ccl17*, *Cxcl2* (a functional homologue of human *Il8*), *Ccl22*, and *Cxcl3*), and chemokine receptors (i.e., *Cmklr1*). Further studies revealed that the anti-inflammatory effects of 10(*Z*)-HDA are mediated by PPARα. Thus, following phagocytosis of heat-killed *M. vaccae* NCTC 11659, the mycobacterial lipid 10(*Z*)-HDA may mimic endogenous ligands of PPARα such as palmitoleic acid [280,281], and the endocannabinoid palmitoylethanolamide (PEA) [282,283], or increase/restore adequate PPARα expression to limit host inflammatory responses. Of note, recent findings suggest that the activation of PPARα may affect resilience, neuronal plasticity, and cognitive functioning in the aftermath of traumatic exposure, as is the case in PTSD [284].

#### 2.2.4. *M. vaccae* ATCC 15483^T^ Effects on CD8^+^ CTL

A study from Skinner and colleagues [74] aimed at analyzing the effects of *M. vaccae* ATCC 15483^T^ on CD8^+^ CTL function against macrophages infected with *M. tuberculosis*. Therefore, *M. vaccae* ATCC 15483^T^ was suspended in PBS at a concentration of 10 mg/mL (equivalent to 10^10^ bacilli per ml), autoclaved (15 min, 120 °C), and a single dose of 1 mg was injected intraperitoneally (i.p.) in specific pathogen-free (SPF) BALB/c mice. CD8^+^ CTL were isolated from the spleen two, three, or four weeks after injection. Another set of syngeneic and non-treated mice were used to isolate peritoneal macrophages for in vitro infection with live *M. tuberculosis* (10^5^ macrophages + 2 × 10^5^ bacilli overnight). CD8^+^ CTL were then co-cultured with infected macrophages to assess their cytotoxic activity. Interestingly, co-culturing of CD8^+^ CTL isolated from spleens of *M. vaccae* ATCC 15483^T^-treated mice with *M. tuberculosis-*infected macrophages revealed that CD8^+^ CTL isolated two weeks, but not three or four weeks following *M. vaccae* ATCC 15483^T^ immunization, were specifically cytotoxic against *M. tuberculosis*–infected, but not uninfected, macrophages. In line with these findings, *M. vaccae* ATCC 15483^T^-primed CD8^+^ T cells produce more IFNγ and, consequently, enhance the production of IL-12 by *M. tuberculosis*-infected macrophages during in vitro co-incubation. This finding is of particular note considering that the interplay between IFNγ and IL-12 is crucial in the *M. tuberculosis* clearance of infected macrophages [285]. Noteworthy, in vitro *M. vaccae* ATCC 15483^T^ pre-incubation of CD8^+^ cytotoxic T cells isolated later than two weeks following the initial in vivo immunization prior to co-incubation with *M. tuberculosis-*infected macrophages re-activated their specific cytotoxic activity, suggesting that *M. vaccae* ATCC 15483^T^ can induce memory CD8^+^ T cells capable of recognizing and killing *M. tuberculosis*-infected macrophages upon re-stimulation.

#### 2.2.5. *M. vaccae* NCTC 11659 Effects on Tregs

Similarly to TB, a shift from a Th2 to a Th1 immune response has proven to also be beneficial against asthma, which is characterized by an increased Th2 over Th1 immune response [211]. However, although s.c. administration of a single dose of *M. vaccae* NCTC 11659 (SRP299, 0.1 mg in 200 µL saline) had protective effects in a mouse model of OVA-induced allergic asthma [40,286], it did not induce a shift from Th2 to Th1 immunity, indicated by the fact that the levels of the Th1 cytokines IFNγ, IL-2, and IL-12 were not different between the groups. Interestingly, and in line with increased levels of IL-5 and IL-10 in cultured splenocytes of *M. vaccae* NCTC 11659^T^-treated mice [40], which correlated with decreased lung eosinophilia, numbers of IL-10 producing Tregs were also elevated following *M. vaccae* NCTC 11659 treatment [40]. As transfer of CD4^+^CD45RB^Lo^ Tregs from *M. vaccae* NCTC 11659-treated mice into asthmatic mice strongly suppressed allergen-induced eosinophilic lung inflammation in recipient mice [40], these studies were the first describing a novel Treg-based mechanism by which *M. vaccae* NCTC 11659 modulates immune function.

Support for a role of Tregs in the allergy protective effects of *M. vaccae* NCTC 11659 comes from Hunt and colleagues [75], who first showed higher IL-10, but not IFNγ and IL-12, concentrations in supernatants of mesenteric lymph node cells (mesLNCs) isolated from naïve mice and cultured in vitro for 72 h in the presence of different doses of *M. vaccae* NCTC 11659 (100, 200, and 400 µg/mL) compared with respective PBS conditions. In follow-up experiments, *M. vaccae* NCTC 11659 (0.1 mg/100 µL of sterile water) was administered intragastric (i.g.) and mesLNCs and splenocytes were isolated one, four, or seven days after administration. After 72 h of culturing in the presence of *M. vaccae* NCTC 11659 (300 µg/mL), splenocyte supernatants showed increased IL-10 and IFNγ, but not IL-12, while mesLNC supernatants did not show variations in those cytokines. Moreover, mice treated with *M. vaccae* NCTC 11659 i.g. (0.1 mg/100 µL) and then immunized with two i.p. injections of OVA after three weeks and challenged with intratracheal OVA after six weeks from treatment showed decreased numbers of cell infiltrates and increased levels of IL-10, but not IL-5, in the broncheoalveolar lavage (BAL) fluid. Interestingly, and opposed to what was observed in the BAL, cultured splenocytes showed increased IL-5 but not IL-10 following in vitro stimulation for 72 h with OVA.

In line with and extending the so far reported studies, Adams and colleagues showed that a single administration of *M. vaccae* NCTC 11659 (s.c.; 100 µL; 1 mg/mL) vs. saline to mice prior (day-21) to OVA immunization (day 8) and OVA challenge (day 19; i.t.) reduced pulmonary inflammation, indexed by a decreased BAL total cell number and increased IL-10 levels [39]. Interestingly, CD11c^+^ cells isolated from the lungs of *M. vaccae* NCTC 11659-treated mice were characterized by increased levels of IL-10, TGFβ1, and IFNα mRNA expression, supporting the above-mentioned data indicating that *M. vaccae* NCTC 11659 is able to facilitate the effect of antigen-presenting DCs to promote Treg differentiation [287,288].

Evidence that the protective effects of *M. vaccae* NCTC 11659 at least in part are mediated by the induction of Tregs also comes from our own studies, in which we administered *M. vaccae* NCTC 11659 repeatedly via the s.c. route prior (days −21, −14, −7) to the start (day 1) of a mouse model of PTSD [33,289]. To induce this PTSD-like phenotype, the chronic subordinate colony housing (CSC) paradigm, which is based on the repeated psychosocial traumatization (i.e., social defeat) in combination with chronic subordination of four male CSC mice toward a dominant resident male conspecific, was used [289]. Briefly, compared with single-housed controls (SHC), CSC mice avoid trauma-related external reminders, indicated by a lack of social preference toward unfamiliar male mice, and develop a long-lasting increase in general anxiety-related behavior and alcohol consumption/preference, hyperactivity, spontaneous colitis, and an aggravated dextran sulfate sodium (DSS)-induced colitis. CSC exposure is further associated with basal hypocorticism, increased dexamethasone suppression of adrenocorticotropic hormone (ACTH), and increased hypothalamic–pituitary–adrenal (HPA) axis reactivity toward novel stressors. Importantly, CSC mice further showed functional glucocorticoid resistance of isolated and LPS-stimulated splenocytes and reduced mesLNC Treg counts, together contributing to an overall increased systemic inflammatory state [289]. Importantly, repeated s.c administration of a heat-killed preparation of *M. vaccae* NCTC 11659 (0.1 mg in 100 µL) induced a shift toward proactive stress coping, prevented/ameliorated CSC-induced anxiety, social anxiety, spontaneous colitis, and aggravation of DSS-induced colitis [33]. As reported at the beginning of this section in a mouse model of airway inflammation [40], *M. vaccae* NCTC 11659 propagated its immunoregulatory and, thus, PTSD-protective effects via induction of Tregs and IL-10 secretion [33]. The latter was indicated by the fact that pretreatment with an anti-CD25 antibody, which depletes Tregs, but not pretreatment with a control-antibody, prevented the stress-protective effects of prior *M. vaccae* NCTC 11659 immunization.

#### 2.2.6. *M. vaccae* NCTC 11659 Effects on Brain Microglia

In addition to the well-documented immunomodulatory effects of *M. vaccae* NCTC 11659 on peripheral immune functioning, data suggest that immunization with *M. vaccae* NCTC 11659 also has the potential to attenuate stress-induced neuroinflammation. For example, immunization of adult male Sprague Dawley rats with *M. vaccae* NCTC 11659 (s.c., 0.1 mg in 0.1 mL sterile BBS) on days –21, –14, and –7, prevents inescapable stress-induced increases in anxiety-like defensive behavioral responses assessed 24 h following stress exposure in a model of learned helplessness [24]. This effect is associated with *M. vaccae* NCTC 11659-induced increases in the expression of hippocampal IL-4 mRNA and protein, which has anti-inflammatory effects in the central nervous system and has been shown to induce anxiolytic and antidepressant-like behavioral responses following central administration [290,291]. Supporting effects of *M. vaccae* NCTC 11659 on anti-inflammatory signaling in the central nervous system, immunization of rats with *M. vaccae* NCTC 11659 increases expression of IL-4-responsive genes including *Cd200r1* and mannose receptor C-type 1 (*Mrc1*; *Cd206*). Cd200r1 is the cognate receptor for Cd200, which inhibits microglial function. In line with a direct effect of *M. vaccae* NCTC 11659 on microglia, immunization of rats with this “old friend” prevented stress-induced microglial priming [24]. Specifically, immunization with *M. vaccae* NCTC 11659 prevented stress-induced exaggeration of LPS-induced secretion of IL-1β from freshly isolated and cultured hippocampal microglia [24]. Immunization with either *M. vaccae* NCTC 11659 or *M. vaccae* ATCC 15483^T^ (both given s.c., 0.1 mg in 0.1 mL sterile BBS on days –21, –14, and –7) has been shown to prevent stress-induced increases in hippocampal *Il6* mRNA expression in adult male Sprague Dawley rats, suggesting that both strains can induce anti-inflammatory signaling in the central nervous system [29]. Consistent with these findings, immunization with *M. vaccae* NCTC 11659 (s.c., 0.1 mg in 0.1 mL sterile BBS on days –19, –12, and –5 prior to laparotomy) increases hippocampal IL-4 and arginase 1 mRNA expression (a biological signature of alternatively activated, M2-like macrophages) as well as forkhead-box-protein (*Foxp*)*3* mRNA expression (a marker of Tregs) while preventing surgery-induced increases in IL-1β mRNA and protein and stress-induced cognitive impairment in a model of post-operative cognitive function, in aged (24 months) male F344XBN F1 rats [22].

An increasing body of evidence suggests that exaggerated neuroinflammation mediates stress-induced exaggeration of fear learning and stress-induced impairment of fear extinction [229,292,293,294,295,296]. Consistent with these findings, and consistent with the potential for *M. vaccae* NCTC 11659 in prevention of stress-induced impairment of fear extinction, repeated immunization of male Sprague Dawley rats with *M. vaccae* NCTC 11659, either before (s.c., 0.1 mg in 0.1 mL sterile BBS on days –35, –28, and –21 before baseline acoustic startle testing) or after (s.c., 0.1 mg in 0.1 mL sterile BBS on day 1, 8, and 15 following fear conditioning; –35, –28, and –21 before fear extinction training), enhances within-session and between-session fear extinction in the fear-potentiated startle model [25,27,28]. Finally, while sleep deprivation has been identified as an important risk factor for development of PTSD, repeated immunization of male C57BL/6N mice with *M. vaccae* NCTC 11659 (s.c., 0.1 mg in 0.1 mL sterile BBS on day –18, –11, and –4 days before the onset of sleep deprivation) has been shown to prevent a stress-induced sleep and behavioral phenotype that shares features with human PTSD [23].

#### 2.2.7. *M. vaccae* ATCC 15483^T^ Effects on Gene Expression in the Context of TB Infection

In order to understand the protective effects of *M. vaccae* ATCC 15483^T^ in the context of TB reviewed in [297], Gong and colleagues aimed at analyzing the expression of an array of genes involved in inflammatory responses in a mouse model of tuberculosis [52]. In their study, mice were infected with live *M. tuberculosis* (strain H37Rv, 5 × 10^5^ CFU via the caudal vein) on day 0, and received intramuscular (i.m.) injections of *M. vaccae* ATCC 15483^T^ (*M. vaccae*^TM^*,* Longcom batch) or vehicle on day 7, 21, and 35 (22.5 μg in 100 μL distilled water). Fifty-two days following the last injection, (day 87), lung and spleen were homogenized and plated for four weeks to check for *M. tuberculosis* colony formation. To check for gene expression, total RNA from PBMCs was isolated. Interestingly, mice that received *M. vaccae* ATCC 15483^T^ developed significantly less *M. tuberculosis* CFU in the spleen and by trend less *M. tuberculosis* CFU in the lungs, suggesting that *M. vaccae* ATCC 15483^T^ hinders *M. tuberculosis* from establishing infections in those organs. In parallel, *M. vaccae* ATCC 15483^T^ induced the upregulation of genes associated with the TNF signaling pathway, NOD-like receptor signaling pathway, TLR signaling pathway, and mitogen-activated protein kinase (MAPK) signaling pathway. Specifically, the expression of TLR2 was enhanced, accompanied by activation of the NF-κB and MAPK signaling pathways and upregulation of Th1 cytokines (TNF, IL-1, IL-6, IL-12, IL-18) and chemokines (C-X-C motif chemokine ligand 2 (CXCL2), monocyte chemoattractant protein (MCP)-1 (also referred to as CC-chemokine ligand (CCL)2)), together promoting clearance of *M. tuberculosis* [298,299,300]. Furthermore, myeloid differentiation primary response 88 (MYD88) innate immune signal transduction adaptor (MyD88), an adaptor protein in the TLR2 and TLR4 signaling pathways [301] that plays a critical role in immune responses against *M. tuberculosis* infections [302,303], was also upregulated in response to *M. vaccae* ATCC 15483^T^ administration.

## 3. The Route of *M. vaccae* NCTC 11659 Administration Affects Its Immunoregulatory Effects

During the past years, *M. vaccae* NCTC 11659 has been administered to humans and animals via different routes. Although the i.d. route was preferably used in humans, and the s.c. route was preferably used in rodents, some studies employed non-invasive mucosal administration of *M. vaccae* NCTC 11659, e.g., oral (per os; p.o.) in humans as well as i.g. and intranasal (i.n.) in rodents. The reason for dedicating a whole section of this review article to this topic is that different routes of administration are likely to result in: (1) presentation of the antigen to different immune cell types; (2) different antigen concentrations available to be presented to the respective immune cells; and (3) different effects on or at least kinetics of the subsequent immune response.

### 3.1. Invasive Route: s.c. Administration of M. vaccae NCTC 11659 and M. vaccae ATCC 15483^T^

The invasive s.c. route provides a stable and long-lasting presence of the injected antigen, serving as a reservoir of bacterial material that is available for antigen presentation to tissue-resident Langerhans DCs for a longer period of time, thereby increasing its immunomodulatory function [32]. The immunoregulatory and anti-inflammatory effects of s.c. administered *M. vaccae* NCTC 11659 are at least in part mediated via the induction of CD4+CD45RB^low^ Type 1 regulatory (Tr1) Tregs or CD4^+^CD25^+^ forkhead box P3 (FoxP3)^+^ Tregs. This was shown in a mouse model of asthma [40,286] as well as in a mouse model of chronic psychosocial stress [33]. Although it is not yet clear for the in vivo condition whether *M. vaccae* NCTC 11659 can increase Treg counts directly via affecting naïve T cells, in vitro studies support the hypothesis that this represents an indirect process involving *M. vaccae* NCTC 11659-primed immunoregulatory CD11c^+^ DCs, which in turn promotes the differentiation of naïve T cells into Tregs [39]. In addition, data on the use of s.c. *M. vaccae* NCTC 11659 in individuals with TB revealed a DC- and TLR2-dependent general switch from Th2 to Th1 immunity, resulting in improved clearance of TB bacilli [126,127,131,142,150]. Studies in mice confirmed the Th2 to Th1 shift induced by s.c. administration of *M. vaccae* NCTC 11659 or *M. vaccae* ATCC 15483^T^ and extended these findings by showing that both mycobacterial strains administered via the s.c. route enhanced the ability of *M. tuberculosis-*infected macrophages to eliminate these intracellular bacilli and enhance the ability of CD8^+^ CTLs to kill infected macrophages [74,138].

Invasive administration of *M. vaccae* NCTC 11659 also has beneficial effects on mood and cognition. For instance, participants diagnosed with non-small cell lung cancer [68,93] and treated with standard drug therapy in combination with i.d. administered *M. vaccae* NCTC 11659 reported improved quality of life, indexed by improved cognitive function and mood, reduced body pain, nausea, and peripheral neuropathy [68], besides better tolerance of drug therapy side effects [94] and increased survival [95]. Follow-up studies in rats revealed that s.c. administration of *M. vaccae* NCTC 11659 in *M. vaccae* NCTC 11659-preimmunized mice is able to induce anti-depressive-like behavior, paralleled by an activation of serotonergic neurons specifically in the interfascicular part of the dorsal raphe nucleus 6 h after administration [21,30]. In line with these stress-protective effects, repeated administration of *M. vaccae* NCTC 11659 via the s.c. route prior to chronic psychosocial stress exposure promoted an active stress-coping style, and in a Treg-dependent manner prevented stress-induced anxiety [20,33] and colitis [33]. Furthermore, repeated administration of *M. vaccae* NCTC 11659 via the s.c. route prevents inescapable stress-induced exaggeration of anxiety-like defensive behavioral responses in a model of learned helplessness [24,29], and enhances fear extinction in the fear-potentiated startle model [25,27]. Finally, repeated administration of *M. vaccae* NCTC 11659 via the s.c. route prevents development of a PTSD-like syndrome following a two-hit stressor of sleep deprivation followed by social defeat in C57BL/6N mice [23].

### 3.2. Non-Invasive Routes of Administration of M. vaccae NCTC 11659

#### 3.2.1. i.n. Administration of *M. vaccae* NCTC 11659

In contrast, via both the i.g. and i.n. routes, the mycobacterial antigens are presented to M cells and DCs in the mucosa [304,305]. The i.n. route, for instance, is well known for its tolerance promoting effects, mediated in the nose-draining lymph node microenvironment (i.e., cervical lymph nodes and pulmonary lymph nodes) via immunoregulatory DCs promoting Treg development [306,307]. In addition to Tregs, intraepithelial CD8^+^ γδ T cells from the respiratory mucosa and from the small intestine are also involved in mucosal tolerance [308,309]. Noteworthy in this context is that antigens administered via the mucosal route are not available for local immune cells for a long time, but are readily washed away or degraded by mucosal enzymes, thereby potentially compromising the efficacy of the treatment.

Interestingly, while it could be shown that the non-invasive i.g. administration of *M. vaccae* NCTC 11659 promotes immunoregulation via an increased secretion of the typical Treg cytokine IL-10 from mesLNC and splenocytes [75], repeated administration of *M. vaccae* NCTC 11659 (100 μg in BBS) via the i.n. route does not affect spleen and mesLN Treg counts in a model of chronic psychosocial stress (Reber et al., unpublished data). Although this was independent of whether *M. vaccae* NCTC 11659 was administered prior to (days –21, –14, –7) or during (days 2, 8, 15) chronic psychosocial stress exposure, it cannot be excluded at the moment that Treg function or other immune cell types with regulatory properties are involved. In fact, several cell types, besides the CD4^+^CD25^+^Foxp3^+^ Treg subset, have been recognized in mice for their regulatory function. Immune cells with immunoregulatory potential include tissue-resident memory cells [310], IL-10-producing DCs [311], CD4^+^ Th2-like cells that produce IL-4 and IL-10, and antagonize the activity of Th1 effector cells [312], CD4^+^CD45RB^low^ Tr1 cells that function through the production of IL-10 [40,313], and CD4^+^ or CD8^+^ T cells producing TGFβ (Th3 cells) [314]. Furthermore, as above-mentioned, intraepithelial CD8^+^ γδ T cells from the respiratory mucosa and from the small intestine have also been suggested to be involved in mucosal tolerance [308,309]. In summary, although i.n.-administered *M. vaccae* NCTC 11659 prevents the aggravating effects of stress on DSS-induced colitis when administered during chronic psychosocial stress exposure and shows at least mild stress protective effects when administered prior to stressor exposure [32], future studies are required to elucidate the exact underlying mechanisms.

#### 3.2.2. i.g./p.o. Administration of M. vaccae NCTC 11659

Studies employing i.g./p.o.-administered *M. vaccae* NCTC 11659 assessed its effects on TB in humans [43,58], pulmonary allergic inflammation in mice [75], and (using *M. vaccae* ATCC 15483^T^) anxiety-related behavior in mice [315]. TB participants receiving standard drug therapy in addition to daily p.o. administration with *M. vaccae* NCTC 11659 for one month showed an ameliorated TB-associated weight loss and inflammation, reduced hepatotoxicity of TB drugs, and an improved clearance of sputum from *M. tuberculosis* [43]. Although the underlying mechanisms were not investigated in this phase III trial, an improved sputum clearance in TB participants suggests that p.o. *M. vaccae* NCTC 11659 administration promoted Th1-dependent intracellular killing of TB bacilli inside macrophages [138]. In line with this, TB protective effects of p.o.-administered *M. vaccae* NCTC 11659, either a total of 10 capsules [58] or 3 i.d. injections [41] of *M. vaccae* NCTC 11659 together with TB drug therapy improved body weight, TB bacilli clearance, and normalization of ESR to a comparable extent compared with TB drug therapy alone. Cultured monocytes from both p.o. and i.d. treatment groups revealed an increase in Th1 and a decrease in Th2 cytokines, once again confirming the effects of *M. vaccae* NCTC 11659 on Th1/Th2 polarization. The only difference between the groups was that TB participants administered *M. vaccae* NCTC 11659 via the p.o. route, as opposed to the i.d. route, showed no downregulation of TNF secretion from cultured monocytes [41,58]. Although not investigated in detail, the authors suggest that the immunomodulatory effects of p.o. administered *M. vaccae* NCTC 11659 could be mediated by intestinal M cells, which phagocytize mycobacteria and mycobacterial antigens and transport them to macrophages in the epithelium [155,156]. In analogy, Hunt and colleagues [75] aimed at assessing whether the protective effects of i.g. and s.c. administered *M. vaccae* NCTC 11659 differed in a mouse model of allergy. One single dose (0.1 mg/100 µL) of *M. vaccae* NCTC 11659 was administered either i.g. (prevention protocol, day –21) before both OVA sensitization (days 0, 12; i.p.) and OVA challenge (days 19, 21; i.t.), or s.c. (treatment protocol, day 21) during OVA sensitizations (days 0, 12, 42, 54; i.p.) but before OVA challenge (days 61, 63; i.t.). Interestingly and in line with the human data reported above, both the prevention (i.g.) and treatment (s.c.) protocol were comparably effective in reducing pulmonary inflammation by restraining eosinophil infiltration and increasing IL-10 in the BAL. The cytokine environment in the BAL showed a bias toward increased IL-10 production, suggesting for the first time an involvement of Tregs following i.g.-administered *M. vaccae* NCTC 11659, with potentially beneficial consequences for the treatment of allergy. Noteworthy, mice treated p.o. with *M. vaccae* ATCC 15483^T^ via food pellets (4.5 × 10^6^ CFU/mL per food pellet) on days –21 and –7 before behavioral testing in the Hebb-Williams-style complex maze or the elevated zero-maze (EZM) [315] showed a faster maze run time and reduced expression of anxiety-related behavior. Although the neurobiological mechanisms were not elucidated, the authors speculate that the effects of *M. vaccae* ATCC 15483^T^ might be due to its influence on the serotonergic system in the midbrain and pontine raphe nuclei, as shown previously [21].

## 4. Summary and Conclusions

- Preparations of *M. vaccae* NCTC 11659 have been shown, regardless of their administration route, to have immunomodulatory properties (for summary see Figure 1).
- Preparations of *M. vaccae* NCTC 11659 have been shown to be beneficial in a plethora of conditions such as TB, leprosy, psoriasis, dermatitis, allergy, asthma, and several cancers as well as inescapable and chronic psychosocial stress.
- While invasive s.c. and non-invasive i.g. administration of *M. vaccae* NCTC 11659 mediate their protective effects at least in part via induction of Tregs, the non-invasive i.n. administration of *M. vaccae* NCTC 11659 protects against the negative pro-inflammatory consequences of chronic psychosocial stress without affecting splenic and mesLN Treg counts.

## 5. Future Perspectives

Together, data covered in this historical narrative review suggest that combining invasive or non-invasive administration of *M. vaccae* NCTC 11659 with other immunomodulatory substances known to additionally facilitate Treg counts or function in the future might be able to boost the immunoregulatory and, thus, stress-protective effects of *M. vaccae* NCTC 11659. Promising candidates would be *Lactobacillus reuteri* (e.g., WU and 100-23 strains) and retinoic acid (RA). While both substances are well-known for their facilitating effect on the number of Tregs [316,317], RA can, similarly to *M. vaccae* NCTC 11659, induce tolerogenic DCs that facilitate the *de novo* conversion of Foxp3^–^ CD4^+^ cells into Foxp3^+^ Treg cells [317,318]. In addition, in the presence of RA, TGFβ1 inhibits IL-6-dependent Th17 cell formation and promotes Treg development [319,320]. Another *Mycobacterium*, *M. obuense* IMM-101 (NCTC 13365; [321]) has shown, similarly to *M. vaccae* NCTC 11659, remarkable immunomodulatory properties. An interesting concept expressed by Kleen and colleagues [102] states that *M. obuense* IMM-101 as well as other mycobacteria could have pleiotropic effects on the immune system. This concept, also known as “trained immunity”, can be defined as any contact with microbial stimuli that can induce long-lasting epigenetic changes in innate immune cells, which can not only result in enhanced response to a second challenge by the same microbe (immunological memory), but also to unrelated microbial insults [322,323,324]. Interestingly, *M. obuense* IMM-101 has been demonstrated to induce Th1 responses while counter-regulating Th2 responses and showed promising results in clinical trials of melanoma and pancreatic cancer [228,325]. Mechanistically, *M. obuense* IMM-101 enhanced antigen presentation in DCs [72,326], IFNγ production by multiple cell types like natural killer (NK) cells and γδ T cells [71,321] and induced the activation of CD4^+^ Th1 and CD8^+^ CTL [72,321,326,327]. The fact that *M. obuense* IMM-101 has such pleiotropic activity on the innate and adaptive type 1 immune response makes its use an attractive candidate for a therapeutic agent against cancers [228,325] as well as viral infections such as severe acute respiratory syndrome coronavirus type 2 (SARS-CoV-2) [102]. This last statement is supported by data showing that *M. obuense* IMM-101 induces the DC-dependent generation of T cells that secrete the anti-viral molecules IFNγ, perforin, and granzyme B [327,328] and has led to the approval of a Phase 3 trial of immunization with *M. obuense* IMM-101 for the prevention of severe respiratory and coronavirus disease (COVID)-19 related infections in cancer patients [329]. Of note, just like *M. vaccae* NCTC 11659, *M. obuense* IMM-101 is also a rapidly dividing environmental saprophyte [330] and its type strain (DSM 44075^T^, NCTC 10778^T^, ATCC 27023^T^; [330,331]) is highly related to *M. vaccae* NCTC 11659 (99.9% sequence similarity between 16S rRNA genes; [34]). Given the above-mentioned report and the highly similar effects on the immune system between *M. obuense* IMM-101 and *M. vaccae* NCTC 11659, future studies should investigate the immune-training abilities of *M. vaccae* NCTC 11659 against viral infections such as SARS-CoV-2. Combining these two mycobacteria in the same formulation should be at least considered to possibly benefit from their additive/synergistic immunoregulatory effects. Given their anti-inflammatory and immunoregulatory properties, RGMs may be particularly useful for addressing emergent psychiatric conditions associated with SARS-CoV-2 infection [332].

Although more work is needed to fully define the effects of RGM including *M. vaccae* NCTC 11659 on microbiome–gut–immune–brain mechanisms relevant to stress-related psychiatric disorders including PTSD, studies to date support continued research to define the mechanisms involved. In addition, particularly given the extensive use of *M. vaccae* NCTC 11659 in clinical trials and overall safety record, data support phase I or phase I/phase II clinical trials evaluating *M. vaccae* NCTC 11659 for the prevention or treatment of stress-related psychiatric disorders including PTSD. *M. vaccae* NCTC 11659 may have particular promise in an inflammatory subset of individuals, or in individuals with comorbid inflammatory disease and psychiatric symptoms.

## Figures and Tables

**Table 1:** List of alternative names of the mycobacterium strains used in the present review article and related details ^1^.

Reference Strain	Original Source and Year of Isolation	Alternative Collection Numbers	Culture Medium	Batch Name	Inactivation Method	Administration Vehicle	Reference
*Mycobacterium vaccae* NCTC 11659 [34]	Mud (lake Kyoga, Uganda), 1973	- DSM 107316 - CECT 9646	- Peptone meat extract glycerol agar - Middlebrook medium - Löwenstein-Jensen medium - Tryptic Soy Agar - MB7H10 agar - PMG agar - GYM agar	SRP 299	Heat-killed ^2^	PBS	[36,37,38]
						Saline	[39,40]
				SRL172	Heat-killed ^2^	BBS	[41]
					Irradiation-killed ^3,^*	BBS	[42]
				V7	Hydrolyzed and heat-killed ^2^	Oral pill excipients	[43,44]
				Dar-901 (SRL 172)	Heat-killed ^2^	Citrate buffer	[45]
				MV07	Heat-killed ^2^	Culture media	[46]
				MV001	Heat-killed ^2^	BBS *	[47]
				MV007	Heat-killed ^2^	BBS	[48]
				ENG1	Heat-killed ^2^	BBS	[20,22,24,29,32]
				R877R	Irradiation-killed ^3^	BBS	[49]
*Mycobacterium vaccae* ATCC 15483^T^ [50]	Bovine milk, 1964	- ATCC 23004 - CCUG 21003T - DSM 43292 - KCTC 19087 - NCIB 9937 - NCTC 10916 - SN 920 (Bonicke SN920) - TMC 1526	- Middlebrook medium - Medium 55 for *Mycobacterium*	V7	Hydrolyzed and heat-killed ^2^	Oral pill excipients	[51]
				*M. vaccae* ^TM^	Heat-killed ^2^	Distilled water	[52]
				ATCC^®^ 15483	Heat-killed ^2^	PBS	[53]
						BBS	[29]

^1^ Reference strain, original source and year of isolation, alternative collection numbers, culture medium, batch name, administration vehicle and relevant references. ATCC, American Type Culture Collection, Manassas, VA, USA; BBS, borate-buffered saline; CCUG, Culture Collection, University of Göteborg, Department of Clinical Bacteriology, Guldhedsg, Göteborg, Sweden; CETC, Colección Española de Cultivos Tipo; DSM, DSMZ-Deutsche Sammlung von Mikroorganismen und Zellkulturen GmbH, Braunschweig, Germany; KCTC, Korean Collection for Type Cultures, Genetic Resources Center, Korea Research Institute of Bioscience and Biotechnology, Taejon, Republic of Korea; NCIB, National Collection of Industrial Bacteria, Torry Research Station, Aberdeen, Scotland, UK (incorporated with NCIMB); NCTC, National Collection of Type Cultures, Central Public Laboratory Service, London, UK; PBS, phosphate-buffered saline; PMG, Pombe Minimal Glutamate; GYM, glucose, yeast and malt; MB7H10, Middlebrook 7H10; SN, Australian Mycological Panel (AMP; formerly Aust or SN); TMC, Trudeau Mycobacterial Culture Collection, Trudeau Institute, Denver, CO, USA. ^2^ Heat-killed through autoclaving at 121 °C for 15 min (Stanford et al., 1990b). ^3^ Irradiation-killed refers to the sterilization procedure, usually performed through exposure to 2.5 Mrad from a ^60^Co source (Stanford et al., 1990a). * indicates that we did not explicitly find this information in the publication, but deduced it based on other facts given in the publication.

**Table 2:** List of observational studies employing rapidly growing mycobacteria strains (RGMs) in clinical studies ^1^.

Disease Investigated	*M. vaccae* Strain	Dosage	Vehicle	Effects	Underlying Mechanisms	Reference
Tuberculosis	NCTC 11659	1 dose (i.d.; 1 mg in 0.1 mL)	BBS	- improved clearance of TB bacilli - normalized ESR - improved sputum conversion - increased survival	- reduced serum IL-4, IL-10, TNF - increased serum IFNγ	[54,55,56,57]
Tuberculosis	NCTC 11659 (SRL172)	3 doses (i.d.; 1 mg in 0.1 mL)	BBS	- improved clearance of TB bacilli - normalized ESR - improved sputum conversion	increased serum IL-4 & TNF	[41]
Tuberculosis	NCTC 11659 (SRL172)	10 doses (p.o; day 0,7,14,21,28 and then monthly; 1 mg/dose)	Gelatine tablet	- normalized ESR - improved sputum conversion and body weight gain	- increased Th1 parameters - decreased Th2 parameters	[58]
Tuberculosis	NCTC 11659 (SRL172)	5 doses (i.d.; 1 mg in 0.1 mL)	BBS	proliferation of PBMCs	increased IFNγ in PBMCs	[42]
Tuberculosis	NCTC 11659 (V7)	30 doses (p.o.; 10 μg/tablet)	V7 tablet (Immunitor^®^)	improved clearance of TB bacilli	-reduced blood leukocyte number	[43,44]
Tuberculosis	Longcom batch (ATCC 15483^T^)	30 doses (p.o.; 10 μg/tablet)	V7 tablet (Immunitor^®^)	improved clearance of TB bacilli	not investigated	[51]
Leprosy	NCTC 11659	1 dose (i.d.; 0.1 mg in 0.1 mL)	BBS	positive Leprosin A response	not investigated	[59,60]
Leprosy	NCTC 11659	1 dose (i.d.; 1 mg in 0.1 mL)	BBS	improved skin capillary flow	not investigated	[61]
Leprosy	NCTC 11659	3 doses (i.d.; 0.01, 0.1, 1 mg in 0.1 mL)	BBS	positive Leprosin A response	not investigated	[62]
Psoriasis	NCTC 11659	1 dose (i.d.; 1 mg in 0.1 mL)	BBS	reduced PASI	reduced lymphocyte proliferation	[63]
Dermatitis	NCTC 11659 (SRL172)	1 dose (i.d.; 3 mg in 0.3 mL)	BBS	reduced dermatitis lesion area	not investigated	[64]
Asthma	NCTC 11659 (SRL172)	1 dose (i.d.; 1 mg in 0.1 mL)	BBS	trend towards improved airway response to allergen	reduced IL-5 and IgE in PBMCs	[65]
Cancer ^2^	NCTC 11659 (SRL172)	- 1 dose (i.d.; 0.5 mg in 0.1 mL); - 3 doses (i.d.; 1 mg in 0.1 mL)	BBS	increased survival	not investigated	[66]
Cancer ^2^	NCTC 11659 (SRL172)	- 1 dose (i.d.; 50 µL (5 × 10^8^ heat-killed bacilli)); - 4 doses (i.d.; 100 µL (10^9^ heat-killed bacilli))	BBS	improved survival	intracellular IL-2 induction	[67]
Cancer ^3^	NCTC 11659 (SRL172)	5 doses (i.d.; 1 mg in 0.1 mL)	BBS	- improved quality of life (cognitive function and vitality); - reduced body pain, nausea and dyspnea	not investigated	[68]

^1^ Disease investigated, strain of *M. vaccae*, dosage and vehicle used, effects, cellular/molecular mechanisms involved, and respective sources are listed. ^2^ Metastatic malignant melanoma. ^3^ Non-small-cell lung adenocarcinoma. Abbreviations: ATCC 15483^T^, *M. vaccae* American Type Culture Collection 15483^T^; BBS, borate-buffered saline; ESR, erythrocyte sedimentation rate; i.d., intradermal; IFN, interferon; IgE, immunoglobulin E; IL, interleukin; NCTC 11659, *M. vaccae* National Collection of Type Cultures 11659; PASI, psoriasis area severity index; PBMC, peripheral blood mononuclear cells; p.o, per os (i.e., orally); TB, tuberculosis; Th, T helper cell; TNF, tumor necrosis factor.

**Table 3:** List of observational studies employing rapidly growing mycobacteria strains (RGMs) in preclinical studies ^1^.

Disease Investigated	*M. vaccae* Strain	Dosage	Vehicle	Effects	Underlying Mechanisms	Reference
Negative consequences of stress	NCTC 11659	3 doses (s.c.; 0.1 mg in 0.1 mL)	BBS	reduced stress-induced anxiety and colitis	- increased number of Treg (CD4^+^ CD25^+^ FoxP3^+^) - increased IL-10	[20,33]
Negative consequences of stress	NCTC 11659	3 doses (i.n.; 0.1 mg in 0.012 mL)	BBS	reduced stress-induced colitis	not investigated	[32]
Negative consequences of stress	NCTC 11659	3 doses (s.c.; 0.1 mg in 0.1 mL)	BBS	enhanced between- and within-session extinction in fear-potentiated startle paradigm	alteration in serotonergic gene expression	[25,27,28]
Negative consequences of stress	NCTC 11659	3 doses (s.c.; 0.1 mg in 0.1 mL)	BBS	prevention of stress-induced exaggeration of anxiety and microglial priming	- upregulated hippocampal IL4, Cd200r1 and Mrc1 - downregulated hippocampal Nlrp3 and Nfkbia	[24]
Negative consequences of stress	NCTC 11659	3 doses (s.c.; 0.1 mg in 0.1 mL)	BBS	prevention of post-operative cognitive dysfunction	- upregulated hippocampal IL4, Arg1 and Foxp3 - downregulated hippocampal NfκBbia and IL1β	[22]
Negative consequences of stress	NCTC 11659	3 doses (s.c.; 0.1 mg in 0.1 mL)	BBS	prevention of negative outcomes of a two-hit stress models	- prevention of stress-induced decreased Tph2 and Slc6a4 expression - prevention of stress-induced REM sleep disturbances	[23,26]

^1^ Disease investigated, strain of *M. vaccae*, dosage and vehicle used, effects, cellular/molecular mechanisms involved, and respective sources are listed. Abbreviations: *Arg1*, arginase 1 gene; BBS, borate-buffered saline; CD, cluster of differentiation; FoxP3, forkhead box P3; IL, interleukin; i.n., intranasal; NCTC 11659, *M. vaccae* National Collection of Type Cultures 11659; *NfκBbia*: gene encoding nuclear factor of kappa light polypeptide gene enhancer in B-cells inhibitor, alpha; Nlrp3, NLR family pyrin domain containing 3; s.c., subcutaneous; REM, rapid eye movement; Slc6a4, solute carrier family 6 member 4; Tph2, tryptophan hydroxylase 2; Treg, regulatory T cells.

**Table 4:** List of preclinical studies investigating the underlying mechanisms induced by rapidly growing mycobacteria strains (RGMs) ^1^.

Cellular/ Molecular Target Investigated	*M. vaccae* Strain	Dosage	Vehicle	Species	Underlying Mechanisms	Reference
Th1/Th2 balance	NCTC 11659 (MV07)	1, 10, 100 µg/mL (in vitro)	BBS	human (DCs)	- reduced IL-4 - upregulated CD83 and CD86	[46]
Th1/Th2 balance	NCTC 11659 (SRL172)	3 doses (s.c.; 10^6^,10^7^,10^8^ bacteria in 0.1 mL)	BBS	mouse	- reduced serum IgE, IL-4 and IL-5 - reduced eosinophil count in BAL	[69]
Th1/Th2 balance	NCTC 11659	1 dose (s.c.; 10^7^, 10^8^ or 10^9^ bacteria in 0.1 mL)	BBS	mouse	- reduced serum IgE and IL-4 - increased IL-2 in splenocytes	[70]
γδ T cells	NCTC 11659 (SRL172)	100 μg/mL (in vitro)	BBS	human (PBMCs)	upregulated IFNγ, TNF and granzyme B	[71]
CD11b^+^ myeloid cells	NCTC 11659	300 μg/mL (in vitro)	BBS	human (PBMCs)	- downregulated CD62L - upregulated TLR2, TLR4, CD18, CD11a, CD14, CD36, CD44, CD45, CD54 CD58k, CD80, CD86, CD137L, CD206	[72]
CD11c^+^ APC	NCTC 11659 (SRP299)	1 dose (s.c.; 0.1 mg in 0.1 mL)	NaCl	mouse	- decreased cell number in BAL; - increased IL-10+ and TGFβ in lung DCs	[39]
CD14^+^ monocytes	ATCC 15483^T^ (SN920)	in vitro incubation with 1:10 ratio cells:mycobacteria	Medium	human (PBMCs)	increased secretion of TNF and IL-12	[73]
CD8^+^ CTL	ATCC 15483^T^	1 dose (i.p.; 1 mg in 0.1 mL)	PBS	mouse	- increased expression of IFNγ in TB-infected macrophages - increased cytotoxic activity of CTL against TB-infected macrophages	[74]
Tregs	NCTC 11659 (SRP299)	1 dose (s.c.; 0.1 mg in 0.2 mL)	NaCl	mouse	- increased number of Treg (CD4^+^ CD45RB^Lo^) - suppressed airway inflammation upon Treg transfer	[40]
Tregs	NCTC 11659 (SRP299)	- 1 dose (i.g.; 0.1 mg in 0.1 mL) - 100, 200 or 400 μg/mL (in vitro)	-H_2_O-NaCl	mouse	- reduced cellular infiltrate in lungs - increased IL-10 in mesLNC	[75]
Tregs	NCTC 11659	3 doses (s.c.; 0.1 mg in 0.1 mL)	BBS	mouse	- increased number of Treg (CD4^+^ CD25^+^ FoxP3^+^) - increased IL-10 - reduced stress-induced anxiety and colitis	[33]
PPARα	10(*Z*)-HDA from NCTC 11659	200 µM (in vitro)	DMEM/F-12	mouse	PPARα-dependent downregulation of pro-inflammatory transcription factors, cytokines and chemokines	[76]
Serotonergic neurons	NCTC 11659	3 doses (s.c.; 0.1 mg in 0.1 mL)	BBS	mouse	activation of serotonergic neurons in interfascicular part of dorsal raphe nucleus	[21]
Microglia	NCTC 11659	3 doses (s.c.; 0.1 mg in 0.1 mL)	BBS	rat	- increased expression of *Il4* mRNA and IL-4-responsive genes (*Cd200r1, Mrc1*) - reduced IL-1β secretion from freshly isolated and LPS-stimulated hippocampal microglia	[24]
Microglia	NCTC 11659, ATCC 15483^T^	3 doses (s.c.; 0.1 mg in 0.1 mL)	BBS	rat	prevention of stress-induced upregulation of hippocampal *Il6* mRNA expression	[29]
Microglia	NCTC 11659	3 doses (s.c.; 0.1 mg in 0.1 mL)	BBS	rat	increased hippocampal *Il4, Foxp3, Arg1*, decreased *Il1β, Il6 and Nfκbia* mRNA expression	[22]

^1^ Cellular/molecular target investigated, strain of *M. vaccae*, dosage and vehicle used, species, cellular/molecular mechanisms involved, and respective sources are listed. Abbreviations: *Arg1*, arginase 1 gene; ATCC 15483^T^, *M. vaccae* American Type Culture Collection 15483^T^; BAL, bronchoalveolar lavage; BBS, borate-buffered saline; CD, cluster of differentiation; DMEM, Dulbecco’s modified Eagle’s medium; FoxP3, forkhead box P3; HPC, human pancreatic carcinoma cell line, IFN, interferon; i.g., intragastric; IL, interleukin; i.p., intraperitoneal; *Mrc1*, gene encoding mannose receptor C-type 1; NCTC 11659, *M. vaccae* National Collection of Type Cultures 11659; *NfκBbia*: gene encoding nuclear factor of kappa light polypeptide gene enhancer in B-cells inhibitor, alpha; PBMC, peripheral blood mononuclear cells; PBS, phosphate-buffered saline; p.o, per os (i.e., orally); PPARα, peroxisome proliferator-activated receptor α; s.c., subcutaneous; TB, tuberculosis; TNF, tumor necrosis factor.

## Abbreviations

10(*Z*)-HDA	10(*Z*)-hexadecenoic acid
ACTH	adrenocorticotropic hormone
ART	antiretroviral therapy
ATCC	American Type Culture Collection, Manassas, VA, USA
AUC	area under the curve
BAL	bronchoalveolar lavage
BBS	borate-buffered saline
BCG	Bacillus Calmette Guérin
CCL2	C–C motif chemokine ligand 2, also referred to as monocyte chemoattractant protein-1 (MCP-1)
CD	cluster of differentiation
CECT	Colección Española de Cultivos Tipo
CFU	colony-forming units
CCUG	Culture Collection
University of Goteborg	Sweden
CLR	C-type lectin receptor
COVID-19	coronavirus disease 19
CREB	cAMP-response element binding protein
CSC	chronic subordinate colony housing
CTL	cytotoxic T lymphocyte
CXCL2	C-X-C motif chemokine ligand 2
DC	dendritic cell
DSM	DSMZ-Deutsche Sammlung von Mikroorganismen und Zellkulturen GmbH
Braunschweig	Germany
DSS	dextran sulfate sodium
ESR	erythrocyte sedimentation rate
EZM	elevated zero-maze
FoxP3	forkhead box protein P3
GM-CSF	granulocyte-macrophage colony-stimulating factor
GYM	Glucose, yeast, and malt agar
HIV	human immunodeficiency virus
HPA	hypothalamic-pituitary-adrenal
hsp	heat-shock protein
i.d.	intradermal
IFNγ	interferon gamma
i.g.	intragastric
Ig	immunoglobulin
i.m.	intramuscular
i.n.	intranasal
KCTC	Korean Collection of Type Cultures
IL	interleukin
INH	isonicotinic acid hydrazide (isoniazid)
IPT	INH preventative therapy
i.t.	intratracheal
LPS	lipopolysaccharide
MAPK	mitogen-activated protein kinase
MB7H10	middlebrook 7H10 agar
M cells	microfold cells
MCP-1	monocyte chemoattractant protein-1, also referred to as C-C motif chemokine ligand 2 (CCL2)
MDD	major depressive disorder
mesLN	mesenteric lymph nodes
mesLNC	mesenteric lymph node cells
MHC	major histocompatibility complex
*M. kyogaense*	*Mycobacterium kyogaense*
*M. vaccae*	*Mycobacterium vaccae*
MyD88	MYD88 innate immune signal transduction adaptor
NCIB	National Collection of Industrial Bacteria
NCTC	National Collection of Type Cultures, Central Public Laboratory Service, London, UK
NF-κB	nuclear factor-κB
NK	natural killer cell
NKG2D	natural killer group 2D
NLR	nucleotide-binding oligomerization domain (NOD)-like receptors
NOD	nucleotide-binding oligomerization domain
OVA	ovalbumin
PASI	Psoriasis Area Severity Index
PBMC	peripheral blood mononuclear cells
PBS	phosphate-buffered saline
PEA	palmitoylethanolamide
PMG	proteose peptone-meat extract-glycerol agar
p.o.	per os (i.e., orally)
PPARα	peroxisome proliferator-activated receptor alpha
PRR	pattern recognition receptor
PTSD	posttraumatic stress disorder
RA	retinoic acid
RGMs	rapidly growing mycobacteria
SARS-CoV-2	severe acute respiratory syndrome coronavirus type 2
s.c.	subcutaneous
SHC	single-housed control
SN	Australian Mycological Panel
SPF	specific pathogen-free
TB	tuberculosis
TCR	T cell receptor
TGFβ1	transforming growth factor beta 1
Th	T helper cell
TLR	Toll-like receptor
TMC	Trudeau Mycobacterial Culture Collection
TNF	tumor necrosis factor, also referred to as tumor necrosis factor alpha
Treg	regulatory T cells
Veh	vehicle
